# Recent Advances in the Molecular Design and Delivery Technology of mRNA for Vaccination Against Infectious Diseases

**DOI:** 10.3389/fimmu.2022.896958

**Published:** 2022-07-15

**Authors:** Lu Yang, Lin Tang, Ming Zhang, Chaoyong Liu

**Affiliations:** ^1^ College of Life Science and Technology, Beijing University of Chemical Technology, Beijing, China; ^2^ Beijing Advanced Innovation Center for Soft Matter Science and Engineering, Beijing University of Chemical Technology, Beijing, China; ^3^ Department of Pathology, Peking University International Hospital, Beijing, China

**Keywords:** mRNA vaccine, infectious diseases, COVID-19, mRNA structure design, mRNA delivery

## Abstract

Vaccines can prevent many millions of illnesses against infectious diseases and save numerous lives every year. However, traditional vaccines such as inactivated viral and live attenuated vaccines cannot adapt to emerging pandemics due to their time-consuming development. With the global outbreak of the COVID-19 epidemic, the virus continues to evolve and mutate, producing mutants with enhanced transmissibility and virulence; the rapid development of vaccines against such emerging global pandemics becomes more and more critical. In recent years, mRNA vaccines have been of significant interest in combating emerging infectious diseases due to their rapid development and large-scale production advantages. However, their development still suffers from many hurdles such as their safety, cellular delivery, uptake, and response to their manufacturing, logistics, and storage. More efforts are still required to optimize the molecular designs of mRNA molecules with increased protein expression and enhanced structural stability. In addition, a variety of delivery systems are also needed to achieve effective delivery of vaccines. In this review, we highlight the advances in mRNA vaccines against various infectious diseases and discuss the molecular design principles and delivery systems of associated mRNA vaccines. The current state of the clinical application of mRNA vaccine pipelines against various infectious diseases and the challenge, safety, and protective effect of associated vaccines are also discussed.

## Introduction

Vaccines prevent many millions of illnesses and save numerous lives every year. The vaccine is a biological agent that provides active acquired immunity to a specific infectious disease, preventing millions of diseases and saving countless lives each year (1). Vaccination against smallpox in the world is the best example of immunization to eradicate infectious diseases (2). Traditional vaccines, such as inactivated viral vaccines, live attenuated vaccines, subunit vaccines, and recombinant vector vaccines, are the most commonly used vaccines in clinical practice, providing lasting protection for various diseases. Inactivated vaccines cannot induce cytotoxic T lymphocytes (CTL) production by endogenous antigen presentation because inactivated pathogens cannot enter host cells and proliferate, limiting their immune effects. Live attenuated vaccines are at risk of reverting to mutations in the body, and people with immunodeficiency and pregnant women generally cannot receive live attenuated vaccines. The subunit vaccine is safe, effective, and low-cost, and the recombinant vector vaccine can be used as a multivalent vaccine for various protective antigens. Despite these successes, significant hurdles remain in developing vaccines against a variety of infectious diseases, requiring better evasion of adaptive immune responses, faster development, and large-scale production.

In addition, traditional vaccines are not suitable for controlling lethal infectious diseases such as malaria, acquired immunodeficiency syndrome (AIDS), and tuberculosis. The RTS.S (Mosquirix) vaccine developed by GlaxoSmithKline (GSK) is the first malaria vaccine to pass clinical trials. Four doses of the vaccine provide only 30% protection against severe malaria in less than three years (3). The protective effect of RTS.S diminished rapidly over time and was age-dependent. Bacille Calmette-Guérin (BCG) is the only vaccine approved to prevent tuberculosis (4). Although it can significantly reduce the incidence of miliary tuberculosis and tuberculous meningitis in children, its protective power is unstable and variable, precluding its use in immunocompromised patients (5–7). AIDS is a highly harmful infectious disease caused by the human immunodeficiency virus (HIV). Since HIV was first reported in 1981, only five trials have entered phase 3 clinical trials, and an effective vaccine against HIV has not yet been developed (8, 9). In addition, HIV, as an RNA virus, has a high frequency of genome mutation, so vaccines derived from specific evolutionary strains may be ineffective against other evolutionary branches.

**Figure 1:**
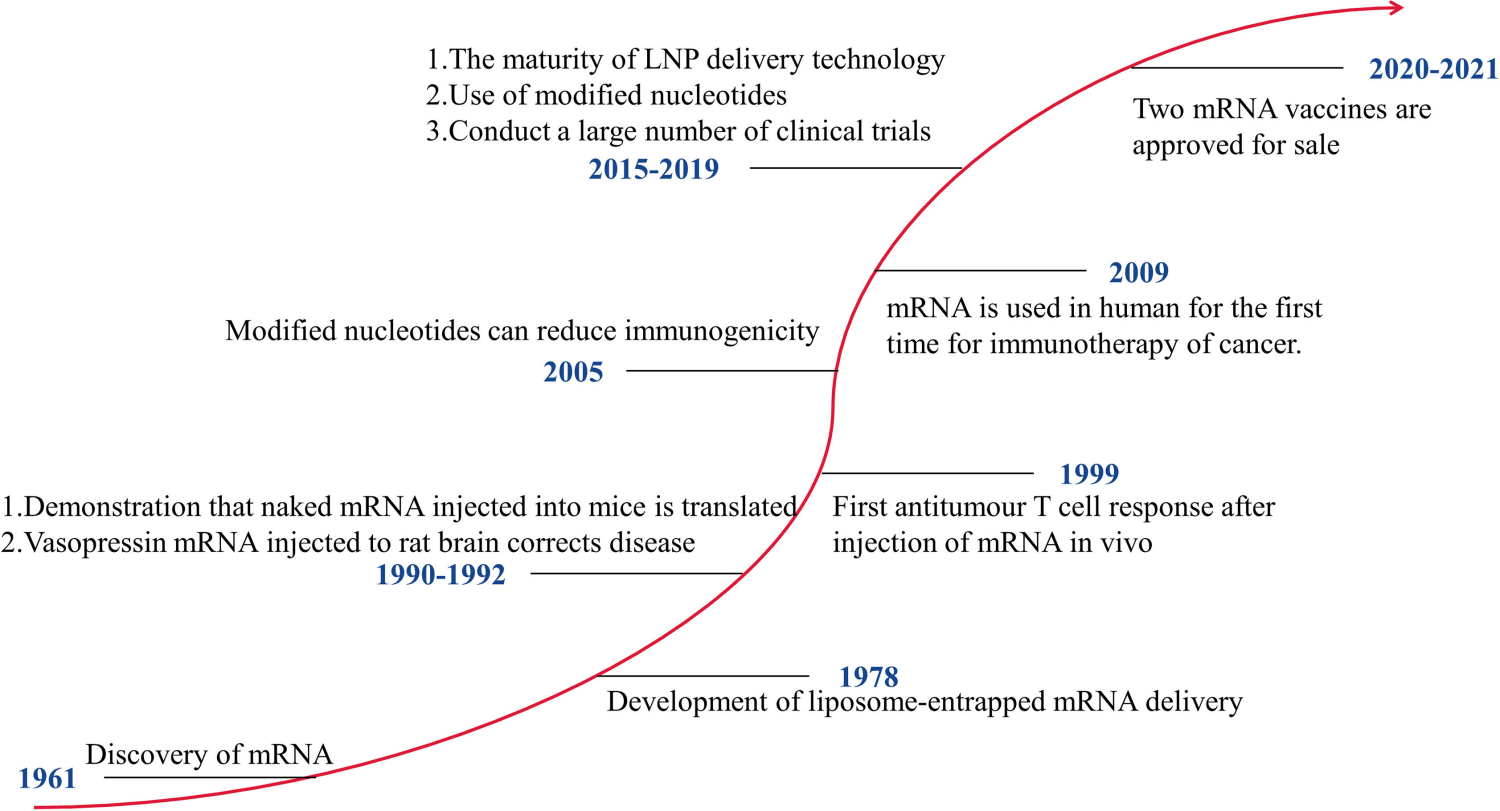
The Development of mRNA Technology.

mRNA vaccines that can be developed rapidly have significant advantages in response to pandemics. **Figure 1** shows the development history of mRNA vaccines. In 1990, mRNA encoding a reporter gene was injected into mice, and the expression of the protein was detected (10). This is the first successful application of *in vitro* transcribed (IVT) mRNA in animal objects. The mRNA vaccines have not received significant investment due to their instability, high inherent immunogenicity, and low *in vivo* delivery efficiency. After 2005, with technological breakthroughs in modified nucleotides and delivery vehicles, mRNA vaccines have developed rapidly, and many clinical trials have been carried out. The outbreak of COVID-19 has made the benefits of mRNA vaccines clinically verified. At present, two mRNA vaccines, mRNA-1273 and BNT162b2, have been put on the market. Of course, getting an mRNA vaccine to market requires a lot of investment. The mRNA-based technology platform covers the mRNA delivery systems and the mRNA transformation technology. In 2019, Moderna and BioNTech spent $496 million and $254 million, respectively, on developing mRNA vaccines. Moderna has spent over $500 million on improving its technology platform and intellectual property.

These results show that mRNA vaccines can overcome the limitations of traditional vaccines and have future development prospects. In this review, we will discuss current mRNA vaccine synthesis methods, summarize recent discoveries in the field, and look forward to the prospects of mRNA vaccines. Compared with the previous reviews, this article not only thoroughly explains the molecular design and optimization of mRNA vaccines, delivery vehicles, and their clinical applications but also summarizes in detail the latest mRNA vaccines against the COVID-19.

## The Molecular Design and Optimization Principles of mRNA Structures

### Pharmacological Properties of mRNA

**Figure 2:**
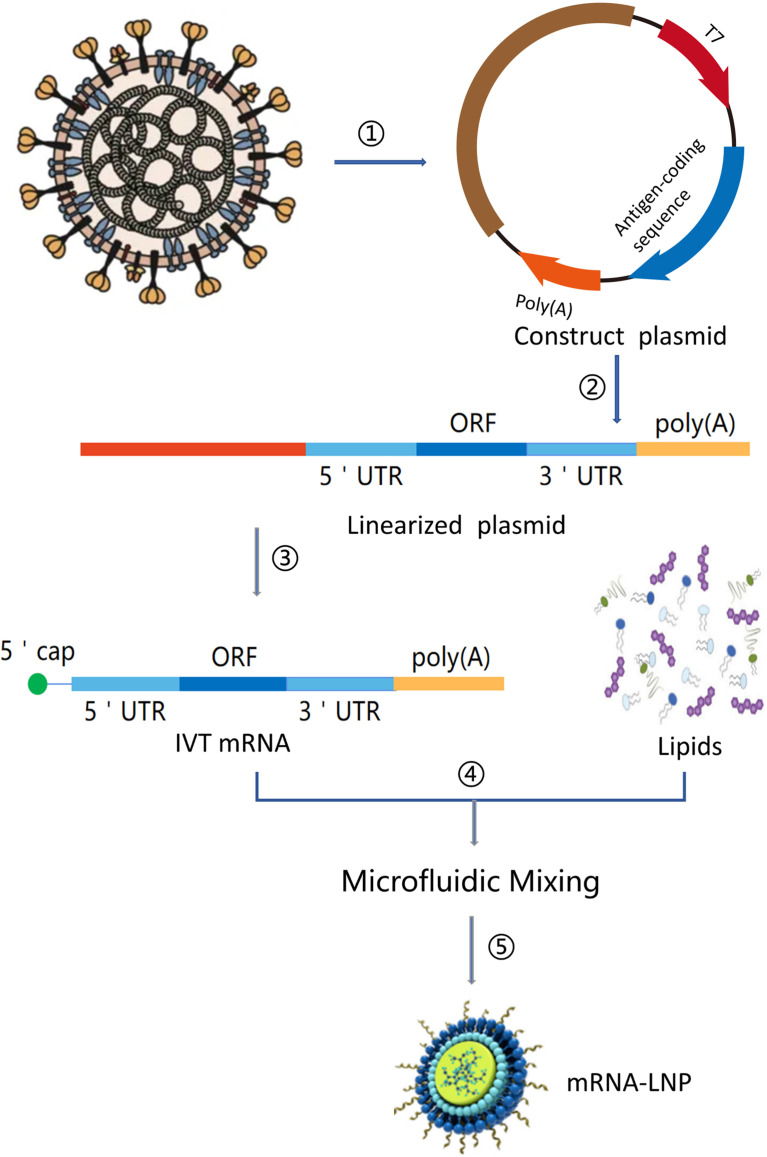
mRNA vaccine manufacturing process. (1) Sequencing and analysis of essential proteins of the virus; (2) The introduction of the plasmid into Escherichia coli and cultured and proliferated; (3) Plasmid extraction, purification, and Enzymatic digestion; (4) *in vitro* transcription of mRNA; (5) Microfluidic mixing; (6) The process of encapsulating mRNA into lipid nanoparticles (LNPs).

mRNA is a single-stranded ribonucleic acid that is transcribed using DNA as a template, and it can carry genetic information to guide protein synthesis. IVT mRNA-based therapy has the following advantages (11): (1) There is no risk of infection or insertion mutation of mRNA. In addition, mRNA can be wholly degraded through the physiological metabolic pathway; (2) mRNA can accurately control the expression degree and duration of the encoded protein; (3) mRNA has a high transcription yield *in vitro* and has the potential for rapid, cheap, and large-scale production. At present, there are two main types of mRNA for drug research: non-replicating mRNA and self-amplified mRNA, from virus sources. The best structure contains 5’cap, untranslated regions (UTRs), the poly(A) tail, and the open reading frame (ORF) encoding target antigen (12)(**Figure 2**). The difference between self-amplified mRNA and non-replicating mRNA is that the ORF of self-amplified mRNA not only encodes the target antigen, but also produces a viral replication mechanism that makes intracellular RNA self-amplify and increases protein expression, and the full length of self-amplified mRNA is much larger than that of non-replicating mRNA (≈ 9-10kb) (13).

### Translation Efficiency and Stability of mRNA

The stability and translation of mRNA are governed by a complex network of RNA/protein interactions and depend on both the primary and secondary structure of mRNAs, translation rates, and degradation mechanisms (11). A lot of efforts have been made to modify the composition of IVT mRNA to systematically improve its intracellular stability and translation efficiency, especially the 5’ cap, 5’,3’ UTR, ORF, Poly (A) tail, and nucleotides (14). These increase the expression of encoded proteins from minutes to weeks (15, 16).

#### The 5’Cap

The cap structure can enhance the translation of mRNA. Combining a 5’ cap with eukaryotic translation initiation factor 4e (EIF4E) is the key to effective translation (17). In Chinese hamster ovary (CHO) cells, the addition of a cap increased the translation of mRNA without poly (A) tail by 2.7 times (18). However, the addition of cap analogues has reverse integration, that is, the cap is integrated into the mRNA in the opposite direction. Take the cap analogue m7GpppG, which is the most commonly used in clinical trials, as an example, the m7G part of m7GpppG is connected to the first nucleotide residue of the RNA chain through a 3’ -5’ phosphodiester bond (19). Pasquinelli et al. (20)found that the reverse integration of m7 GpppG, a cap analogue from 1/3 to 1/2, into mRNA, decreased the translation activity of mRNA because it could not be recognized by translation mechanisms. Therefore, the anti-reversal cap analogue (ARCAs; m_2_^7,3’−O^GpppG) is proposed, ensuring 100% correct direction (21). In various cell types, ARCA-capped mRNAs have better translation efficiency. For example, in rabbit reticulocyte lysate, the translation efficiency of the ARCA capped transcript is 2.3-2.6 times higher than that of m7GpppG (19).

Also, Cap is one of the determinants of mRNA degradation. It regulates the degradation of mRNA, in combination with scavenger decapping enzyme Dcp1, Dcp2, and DcpS (17). There are two types of capping enzymes in eukaryotic cells, which have different biological functions, substrate specificity and regioselectivity of hydrolysis (22). The first enzyme, the complex of Dcp1 and Dcp2 (Dcp1/Dcp2), is involved in the 5’→3’mRNA degradation pathway (17). Dcp2 is the catalytic subunit of Dcp1/Dcp2, which cleaves the cap between α and β phosphate esters, releasing m7GDP and 5’ -phosphorylated mRNA chains and exposing them to the digestion of exonuclease xrn1 (23, 24). DCPS is the second degrading enzyme, which is involved in the 3’→5’ mRNA degradation pathway, selectively cleaving the cap between the β and γ phosphates of the 5’,5’-triphosphate bridge, and then releasing m7GMP and a downstream (oligo) nucleotides (17). Grudzien-Nogalska, E. et al. (25)recently synthesized a series of dinucleotide cap analogues containing phosphorothioate groups at the α, β or γ position of the 5’-5’-triphosphate chain, which included m_2_^7,2’-O^GpppSG, m_2_^7,2’-O^GppSpG, and m_2_^7,2’-O^GpSppG, respectively. Only m_2_^7,2’-O^GppSpG D2 has complete resistance to Human Decapping Scavenger(hDcp)2, while m_2_^7,2’-O^GppSpG D1 and m_2_^7,2’-O^GpppSG have only partial resistance to hDcp2. In cultured HC11 mammary epithelial cells, the t_1/2_ of m7GpppG-capped luciferase mRNA was 86 min, while the t_1/2_ of luciferase mRNA was capped with m_2_^7,2’-O^GppSpG (D2) was 257 min, capping further prolongs the half-life of mRNA (21).

#### The Poly(A) Tail

The poly(A) tail regulates the stability and translation effectiveness of the mRNA in cooperation with cap, internal ribosome entry sites, and other determinants (18). Sachs and Davis (26) proposed that the interaction between poly (A) tail (regulated by poly (A) binding protein) and 60s subunit is essential in controlling the formation of the 80s initiation complex. Because of their dual regulation, the translation efficiency and stability of mRNA have been improved by adding a cap or Poly (A) tail. The expression and half-life of luciferase in uncapped tobacco protoplasts with poly (A) Luciferase (Luc) mRNA were 1.5 and 1.4 times higher than those without poly (A), respectively (18). However, in capped mRNA, the expression of mRNA with poly (A) tail was 21 times higher than that of mRNA without poly (A) (18). Analysis in Dendritic Cells (DCs) showed that a poly (A) tail should not be added at the 3’ end. The optimal length of the masked poly (A) tail should be between 120 and 150 nucleotides (27, 28).

#### 5’- and 3’-UTRs

The 5’ and 3’ UTRs elements on both sides of the coding sequence significantly affect the stability and translation of mRNA (29). Studies have shown that the 5’- end unstructured area should not contain upstream open reading frames, which can avoid incorrect translation initiation and replacement of reading frames (30). Introducing an optimized Kozak sequence (GCCRCCAUGG, vertebrates) can also avoid incorrect startup (31). It has been reported that because stable secondary structures block small ribosome binding initiation coding elements, the 5’UTR region must be short and loose (32). The most common stable pattern is that 3’UTR is rich in pyrimidines, which are recognized by ubiquitous α-complexes (33). The most characteristic sequence recognized by α-complex is about 180bp or about 80bp UTRs in β or α globin, respectively (34). In some preclinical studies, the 3’UTR of IVT mRNA is derived from the 3’UTR of human or African Xenopus β-globin genes (15). Compared with the reporter gene RNA which does not contain UTRs, the protein yield of RNA containing human β-globin 3’UTR is increased (35). Based on the analysis of the experimental data, Holtkamp, S. et al. concluded that the stabilization of the human-globin-UTR sequence was further enhanced by using two human β-globin 3’- UTR arrays in the head-tail (head-to-tail) direction (36). This is mainly because the 3’ UTR of α and β globin is rich in discontinuous pyrimidines (TC) and the corresponding ribonucleoprotein(RNP). RNP can directly interact with poly(A)-binding protein(PABP) to enhance the interaction with the poly(A) tail, protecting the mRNA from degradation. More importantly, there is a stable nucleolar protein binding element in the 3’UTR of β-globin mRNA (37). In addition, the base after terminating the codon is recommended to be G or An to effectively terminate the translation (38). In addition, the 3’- UTR of eukaryotic elongation factor 1 α mRNA (EEF1A1 mRNA) and the 5’- UTR element presenting in many orthopoxvirus mRNAs can not only inhibit capping but also inhibit the extranuclear degradation of 3’UTR, ultimately enhancing the stability and translation efficiency of mRNA (39, 40). To limit the duration of protein production, AU-rich elements can be added to 3’-UTR to rapidly degrade mRNA and shorten protein expression duration (41).

#### The Open Reading Frame (ORF)

The composition of codons will affect the efficiency of translation. Using frequent synonymous codons instead of rare codons can improve translation efficiency because using the same tRNA can speed up translation (42). Mammalian codons usually have G or C nucleotides at the third degenerate position, which is more effectively expressed than codons that end with A or T (43). In this case, it is difficult to distinguish whether the increased expression of codon-optimized proteins is caused by enhanced transcription, increased RNA stability, or enhanced translation. By direct comparison of optimized tRNA usage and removal of RNA secondary structure, potential splicing motifs, and other wild-type sequences in the mRNA, it is found that the resulting protein increased slightly (1.6-3 times) (44, 45). In addition, the use of the most GC-rich codon per codon is another optimization scheme, which can not only increase mRNA stability (*in vitro*) *(* 46) but also increase pr translation (*in vivo*) (47).

Altering codon composition or introducing modified nucleosides (discussed below) can positively regulate protein expression, but may also affect mRNA secondary structure (GC content is related to folding energy) (48), translation dynamics and accuracy, and simultaneous protein folding (49, 50). The use of different synonymous codons affects translation dynamics, leading to different co-translational folding trajectories and ultimately to varying conformations of proteins. The non-random and non-uniform distribution of codons in the gene open reading framework provides a unique biospecific model that regulates local translation elongation and contributes to the stability of mRNA.

#### Modification of Nucleotides

Exogenous mRNA is an innate immune stimulator as it is recognized by various innate immune receptors located on the cell surface, endosome, and cytoplasm (51). The activation of RNA receptors can directly or indirectly affect translation. IVT RNA activates RNA–dependent protein kinase R (phosphorylated translation initiation factor 2-α) and 2-methyl-5-adenylate synthetase (when binding to the target RNA, OAS is activated to produce 2-methyl-5-linked oligoadenylate (2-5A), which activates RNase L, and then RNase L cleaves single-stranded autologous and non-autologous RNA), directly inhibits protein synthesis (52–54). Activation of other cytoplasmic and endosomal RNA sensors, including TLR family (TLR 3, TLR 7, TLR 8), RIG-I, MDA5, NOD2, IFIT-2, DHX9, DDX family and their complex, HMGB protein, and LRRFIP1, can lead to the release of type I interferon, which activates interferon-inducible genes including protein kinase R, RNase L, 2-methyl-5-adenylate synthetase and others, and inhibits translation (14).

By integrating modified nucleotides into IVT mRNA, such as pseudouridine, 2-thiouridine, 5-methyluridine, 5-methylcytidine, or N6-methyladenosine, RNA receptors can avoid being activated to inhibit the immunostimulatory effect of RNA and enhance translation (55–58). For example, Kariko, K. et al. (55) found that the translation of mRNA modified with pseudouridine in 293 cells was ten times higher than that of mRNA without modified nucleotide. The translation of mRNA modified with 5-methylcytidine was four times higher than that of unmodified mRNA.

### Synthesis and Quality Control of IVT mRNA

*In vitro* production of mRNA from linear DNA templates in a cell-free system using RNA polymerases derived from T7, T3, or Sp6 phage (59) (**Figure 2**). This linear DNA template encodes all the structures of the functional mRNA, except for 5’cap. The mRNA can then be capped with enzymes. One way to add cap is to use capping enzyme capping from recombinant vaccinia virus after the initial synthesis of mRNA (60). The resulting cap structure is the same as the most common naturally occurring cap. Another more commonly used method is to add synthetic cap analogues to the transcriptional reaction, cap capping, and *in vitro* synthesis. However, the *in vitro* synthesis of mRNA competes with the cap analogue GTP, which results in the uncapped part of mRNA and the inactivation of translation. Similarly, the poly (A) tail can be transcribed directly from the template vector, or IVT mRNA can be lengthened by recombinant poly (A) polymerase through a two-step reaction. Recombinant poly (A) polymerase can integrate the modified nucleotides into the poly (A) tail and inhibit its deadenylation by poly (A) specific nuclease. One of the limitations of enzymatic polyadenylate is the production of mRNA with poly (A) tails of different lengths. On the contrary, transcribing RNA from a DNA template *in vitro* produces RNA with the same length of poly (A) tail. After that, the DNA template can be degraded by DNA enzyme, and the RNA can be separated by the traditional method of isolating nucleic acid. The 1μg DNA template is sufficient to produce 50-100 μg RNA (59). mRNA synthesized by the enzymatic method contains dsRNA contaminants as abnormal products of IVT reaction, and the presence of pseudouridine and/or M5C cannot eliminate its immunogenicity. high-performance liquid chromatography (HPLC). purification method can be used to eliminate dsRNA contaminants so that mRNA containing modified nucleotides can no longer induce interferon and inflammatory cytokines, and the translation level in primary cells can be increased by 10-1000 times (56). All enzymes and reactive components needed for mRNA’s good manufacturing practice (GMP) production can be expressed as synthetic chemicals or bacteria from commercial suppliers in the form of non-animal component reagents, thus avoiding the uncertainties surrounding cell culture-based vaccine production. All components, such as plasmid DNA, phage polymerase, capping enzyme, and NTPs, can be easily obtained as GMP-level traceable components.

## Delivery Technology of mRNA

**Figure 3:**
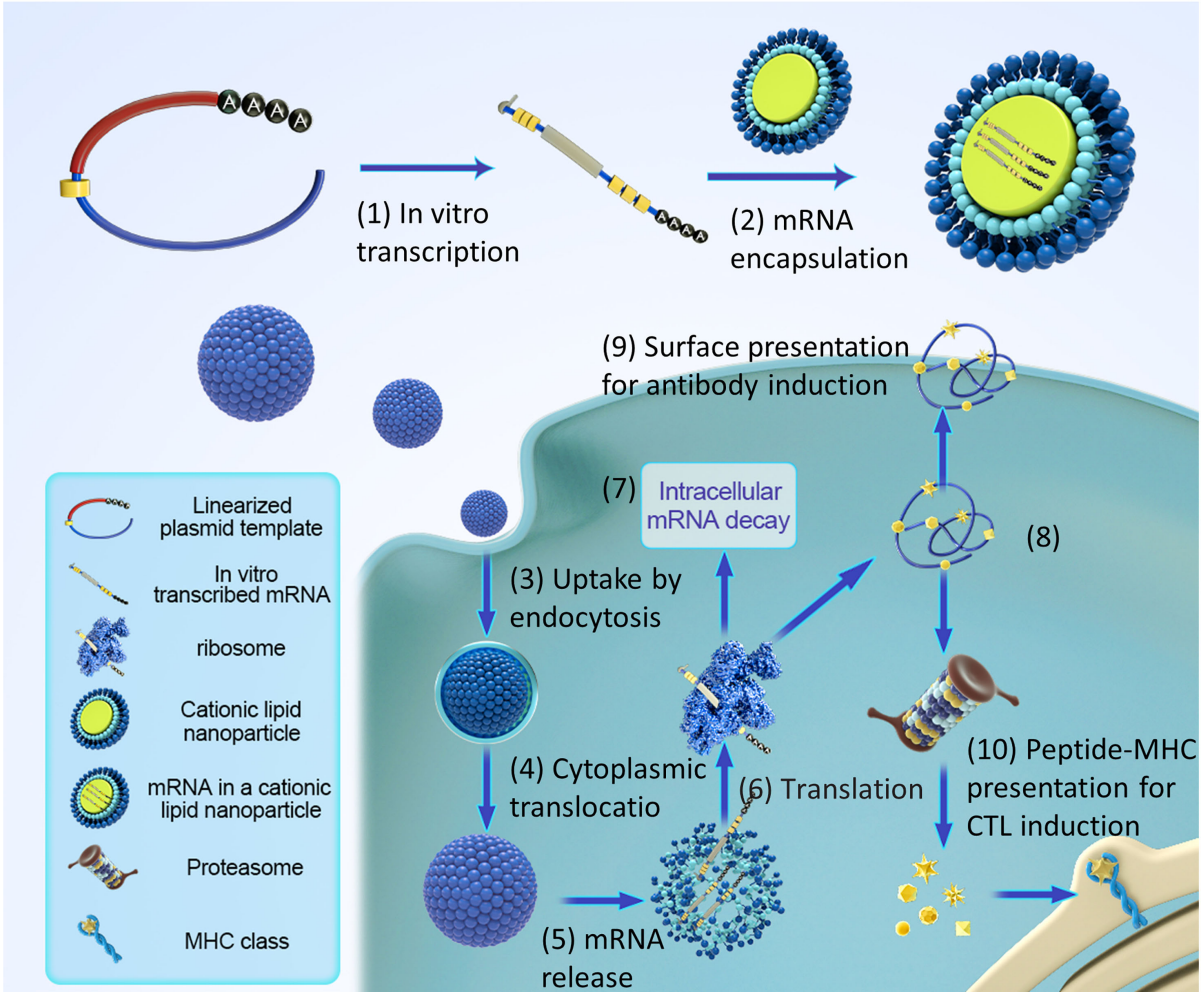
Pharmacology principles of antigen-encoding mRNA. (1) *In vitro* transcription using linearized DNA plasmid templates with antigen coding sequences. The *in vitro* transcribed mRNA contains the structures: cap, 5′and 3′ untranslated regions (UTRs), the open reading frame (ORF), and the poly(A) tail. (2) The synthesized mRNA was compounded with cationic liposomes to form an mRNA-LNP complex. (3) Liposomes protect mRNA from RNase degradation and facilitate cellular uptake of mRNA. (4) Release of the mRNA-LNP complex in the cytoplasm. (5) Release of mRNA from liposome complex. (6) mRNA is translated using the host cell’s protein synthesis machinery. (7) terminates translation by mRNAs degradation catalyzed by an external ribozyme. (8) the post-translational modification of the translated protein product depends on the nature of the host cell. (9) protein-derived epitopes can be presented by MHCI and MHCII molecules on the cell surface. (10) the antigenic peptide epitopes degraded by protein products were loaded on the major histocompatibility complex (MHC) and presented to cytotoxic T lymphocytes.

**Figure 4:**
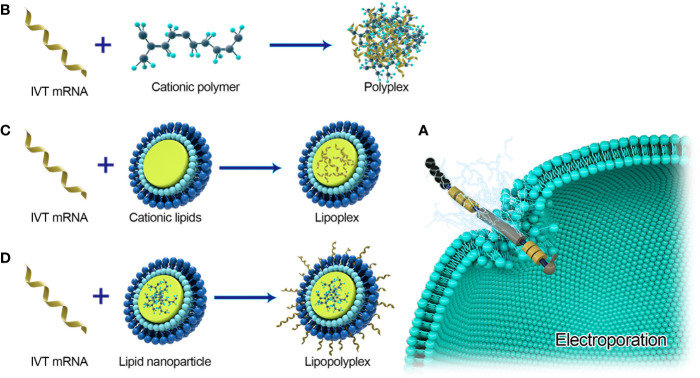
Major delivery systems for mRNA vaccines. **(A)** Electroporation for naked mRNA delivery. **(B)** Polyplexes are formed by spontaneously interacting with negatively charged IVT-mRNA and cationic lipids. **(C)** Lipoplexs formed by loading mRNA into cationic lipids. **(D)** Lipopolyplexs are formed by a polymer ‘core’ and a lipid ‘shell’ with IVT-mRNA absorbed on the surface of the nanoparticle.

Effective delivery of mRNA *in vivo* is the key to the realization of treatment. Exogenous mRNA must cross the lipid membrane barrier to reach the cytoplasm to be converted into functional protein (**Figure 3**). Two key factors determine the cytoplasmic bioavailability of IVT mRNA. One is that a large number of highly active RNase, existing in the outer space of cells, can rapidly degrade IVT mRNA. The other is the cell membrane. It prevents negatively charged large RNA molecules from passively entering the cytoplasm. In principle, eukaryotic cells can actively uptake naked mRNA, but the uptake rate and cytoplasmic transfer rate are the lowest in most cell types (< 1/10000) (12). mRNA can be combined with the delivery system to improve the transfer efficiency and protect RNA from being degraded by nuclease. At present, there are three main strategies for mRNA delivery (1): physical methods that instantly destroy the function of a cell membrane barrier; (2) virus-based approaches that utilize naturally occurring biological uptake patterns; (3) nanocarrier-based non-viral vectors that are reasonably designed and easy to prepare (**Figure 4**).

### Physical Methods

Electroporation was first used in gene transfection in 1982 (61) and has been identified as a popular method for *in vitro* mRNA transfection of hematopoietic cells (62). Immunotherapy of IVT or autologous tumor-derived mRNA electroporation transfected DC in cancer patients has been proved to be safe (63–70). In the IB phase trial, patients with advanced III and IV melanoma developed strong CD4+ and CD8+ immune responses to antigens transcribed by IVT mRNA (64, 71). In the *Phase* I trial, 19 of 20 patients with metastatic prostate cancer also responded to developed antigen-specific CD8+T cells. The development of a new type of equipment for mild electroporation of a large number of cells under aseptic conditions makes it possible to develop a rapid clinical-level scheme for a wide range of cell therapy applications based on IVT mRNA. *In vivo*, electroporation is also used to increase therapeutic RNA uptake. However, in a study by Johansson et al. (72), electroporation only increased the immunogenicity of self-amplified RNA, not non-replicating mRNA. P Qiu et al. (73) proved that mRNA- gold particles complex can express in tissues by gene bombardment, a microplastic method. Gene gun has been proved to be an effective method for RNA transmission and vaccination in mouse models, but there are no efficacy data in large animals or humans (74–76). Physical methods may be limited by their increased cell death and poor contact with target cells or tissues. Recently, this field has tended to use lipids or polymer-based nanoparticles as effective and multi-functional carriers.

### Virus-Based Approach

Recombinant vector particles are produced by co-transfection of vector genome and packaging construction into packaging cells. Tumor retroviral vectors are the first kind of viral vectors developed, and they are the preferred vectors for *in vitro* transfection of regenerated hematopoietic stem cells (77). Foamy virus (FV) is a subclass of retroviruses that can effectively transfect genes into neural progenitor cells and embryonic stem cells. FV vectors mainly target the gene transfection of hematopoietic stem cells (HSCs) (78–80). FV has an extensive tendency, mainly through the interaction between FV glycoprotein and heparan sulfate and the additional unknown receptor molecules on the surface of target cells. Although the effective transduction of the FV vector requires mitosis of host cells, the long-term latent survival of interphase cells also allows effective gene transfection to target tissues with slow division, such as hematopoietic stem cells, due to the highly stable capsid. PFV (prototype FV) vectors seem to increase their translation in transducing cells by at least 10 times. S Ferrari et al. (81) demonstrated that gene transfer mediated by recombinant Sendai virus (SeV) to differentiated airway epithelial cells has been proved to be very effective because of its ability to overcome intracellular and extracellular barriers that limit gene transmission. The virus can spread, so it is unlikely suitable for clinical trials. Viral vectors have inherent defects such as the potential risk of reverse genome insertion, difficulty in controlling gene expression, vector size restrictions, and strong immunological side effects.

### Non-Viral Vector

#### Lipid-Based IVT mRNA Delivery System

Cationic lipids have been the most widely used and studied non-viral vectors for IVT-mRNA transfection. They can spontaneously form liposomes loaded with IVT-mRNA by electrostatic interaction. These lipid-based materials are artificially divided into three categories, DOTAP-based preparations, commercially available liposome preparations, and lipid nanoparticles (LNPs) developed by combinatorial synthesis methodology and library screening.

##### DOTAP-Based Preparations

N-[1-(2-mine3-dienoxy) propyl]-N-trimethyl ammonium chloride (DOTMA) is the first synthetic cationic lipid for *in vitro* delivery of luciferase-coding IVT-mRNA to different cell lines (82). 1,2-dioleoyloxy-3-trimethylammoniumpropane (DOTAP) is a derivate of DOTMA, has relatively low preparation cost and high IVT-mRNA transfer efficiency. Zohra et al. (83) modified DOTAP-based liposome with carbonate apatite (an inorganic crystal with a strong affinity for nucleic acid). Compared with the control group (only DOTAP), the transfection efficiency was increased by 5-15 times. Compared with non-targeted particles, IVT-mRNA-loaded DOTAP- apatite particles incorporated with fibronectin (the recognition motif of α5β1 integrin) could enhance the gene expression of transmitted IVT-mRNA in HeLa cells (84). Another strategy to improve DOTAP-mediated transfection of IVT-mRNA is the addition of neutral lipids to improve the endosome escape of liposomes. DOPE (1-dioleoyl-sn- glycerol-3-phosphate ethanolamine) is one of the most commonly used compounds to enhance gene expression when co-used with cationic lipids (85). The role of this DOPE is attributed to its ability to promote liposome formation and its tendency to transfer liposomes from bilayer structure to hexagonal arrangement at acidic pH levels, which may contribute to intimal fusion or instability (86). Besides, DOPE can also be used as an auxiliary lipid of DOTAP-based liposomes to reduce lipid aggregation (87).

However, the positive charge of cationic lipids hinders their *in vivo* application and clinical development. The positive charge of cationic lipids would promote non-specific interactions with non-target components, such as negatively charged serum proteins, resulting in the rapid clearance of formed aggregates (88, 89). The most common way to overcome this is to use hydrophilic and uncharged polymers, such as polyethylene glycol (PEG), to form a protective film on the surface of nanoparticles. Moreover, the PEG can be further modified with some active targeting ligands. Wang et al. (90) demonstrated PEG and ligand could be incorporated into lipid-based IVT-mRNA delivery systems for tumor therapy. In this study, DOTAP/cholesterol liposome and mRNA-protamine complex were mixed to form a core/membrane complex, and then the complex was coated with 1,2-distearoyl-phosphatidylethanolamine-polyethylene glycol(DSPE-PEG) and DSPE-PEG- anisamide to form liposome-protamine-RNA (LPR) (90). Hydrophilic PEG molecules are expected to block the positive charge of DOTAP/cholesterol lipid bilayers, and anisamide can promote nanoparticles to enter cancer cells with high expression of Sigma receptors through receptor-mediated endocytosis (90). The results show that LPR can transfect up to 68-78% of genes in single cells and mice, and produce more luciferase (25 pg/mg) in tumors than in other tissues (0-2 pg/mg) (90).

##### Commercially Available Liposome Preparations

Commercially available liposomes, such as Lipofectamine and Dreamfect Gold, have been proved to be effective vectors for IVT-mRNA transfection by Balmayor et al. (91). It was observed that Lipofectamine 2000 showed the highest transfection efficiency at 24h. Although the efficiency of DF-Gold was slightly lower than that of Lipofectamine 2000 at 24 h, it could still maintain the expression of MetLuc up to 120 h. However, the compound of MetLuc cmRNA and Lipofectamine 2000 was the most toxic in stem cells, and the cell survival rate decreased to less than 75% within 5 hours (91). The compound of DF-Gold and bPEI had mild cytotoxicity, and the cell survival rate was more than 80% at both time points. In the study by Johler SM et al. (92), the transfection rate of the complex formed by Lipofectamine 2000 and IVT-mRNA encoding green fluorescent protein in human bronchial epithelial cells was more than 50%, while the number of green fluorescent protein-positive cells obtained by polymer-based preparations was 3%. The use of these reagents *in vivo* is limited, in part because of their poor toxicity and transfection ability.

##### Lipid Nanoparticles (LNPs)

LNPs consist of cholesterol (which helps stabilize), natural phospholipids (supporting lipid bilayers), PEG derivatives (reducing aggregation and non-specific uptake), and ionizable liposomes (recombination with negatively charged RNA and increased endosome escape) (93). Researchers at the Novartis Institute used an ionizable lipid DLinDMA (1-diaminophenoxy-3-dimethylpropane) as the main component of LNPs for self-amplification-mRNA vaccination of respiratory syncytial virus fusion glycoprotein (RSV-F). It was prepared by ethanol dilution method, and the particle size ranged from 79 to 121 nm. This vaccine technique with an encapsulation rate of 85%-98% elicits a broad, potent, and protective immune response in mice (94). Anderson and his colleagues have demonstrated that C12-200LNPs, initially developed for siRNA delivery, can deliver IVT-mRNA by optimizing LNP formulation parameters such as lipid weight ratio, phospholipid properties, and excipient composition. In addition, the *in vivo* titer of C12-200LNPs loaded with IVT-mRNA is more than 7 times higher than that of the original preparation (95). Increasing the weight ratio of ionizable lipids to mRNA and adding drugs are considered key features of optimizing prescription. It has been proved that the preparation based on C14-113LNP is an effective non-viral agent, which can efficiently deliver IVT-mRNA to the hearts of small and large animals through multiple administration pathways. The used dose of IVT-mRNA is several orders of magnitude lower than that of previous studies (96, 97). It is speculated that ionized lipids are neutral at physiological pH but positively charged in acidic endosomes. By destroying the membrane of endosomes, ionizable lipids promote the rapid release of RNA goods in mature endosomes, resulting in effective transfection (98).

#### Polymer-Based IVT mRNA Delivery System

Cationic polymers can not only bind IVT-mRNA but also condense it into nanostructures, increase its uptake through endocytosis, protect it from nuclease degradation, and promote its endosome escape. The first trial of polymer-based IVT-mRNA delivery systems can be traced back to 1973 when diethylaminoethyl dextran was used to synthesize IVT-mRNA for *in vitro* transfection (99). Bettinger et al. (99) proved that high molecular weight polymers bind to IVT-mRNA are too close to release, resulting in poor gene expression. In contrast, the expression of the polymer formed with smaller PEI (polyvinyl imine) 2kDa and PLL (poly (L-lysine)) 3.4kDa was 5 times higher in B16-F10 cells than DOTAP, but depended on chloroquine for transfection activity. Li et al. (100) developed an effective PEI-based intranasal vaccination by combining cationic cyclodextrin-low molecular weight PEI conjugates with IVT-mRNA, which can reversibly open tight junctions and enhance the paracellular delivery of IVT-mRNA to overcome the biological barrier of nasal epithelium, thus minimizing the absorption of toxins in the nasal cavity. A strong immune response was obtained through the high mucosal affinity of cyclodextrin and the good adjuvant effect of cationic PEG polymers (100).

#### Peptide-Based Delivery

Cationic peptides containing cationic and amphiphilic amino groups can deliver mRNA into cells. Cationic peptides and mRNAs form nanocomplexes through electrostatic interactions (101). Helen O. McCarthy et al. designed a fusogenic cell-penetrating peptide containing a repeating arginine-alanine-leucine-alanine (RALA) sequence, it can change conformation through endosomal pH, promotes membrane pore formation and endosomal escape (102). This study shows that the formed nanocomplexes have good stability and no cytotoxicity. In addition, PepFect 14 (PF14), a commercialized cell-penetrating peptide consisting of 21 amino acids, is an efficient mRNA delivery vehicle. Brand et al. (103) complexed PF14 with mRNA to form nanoparticles that could deliver mRNA to ovarian cancer cells. Protamine is a small molecular weight, the arginine-rich protein that contains nuclear localization signals. It can efficiently self-assemble with negatively charged nucleotides to form stable complexes (95). Protamine complexed with mRNA can not only protect mRNA from degradation by RNase in serum (104) but also act as an adjuvant. A study in an animal glioma model showed that protamine and mRNA complexes have favorable antitumor effects (105).

#### Cationic Nano-Emulsion

Squalene-based cationic nanoemulsion, which consists of an oily squalene core and a stabilized lipid shell, has the function of mRNA delivery. MF59 is one of the Food and Drug Administration (FDA)-approved formulations of squalene that can be used as an adjuvant in influenza vaccines. MF59 can drive cells to secrete chemokines at the injection site and promote antigen-presenting cell uptake (106). Marcelo M. Samsa et al. (107) constructed a nanoemulsion composed of squalene, DOTAP, and sorbitan trioleate, and used this preparation to deliver mRNA, which has a robust immune effect.

#### Hybrid-Based IVT mRNA Delivery System

IVT-mRNA can be loaded into hybrid nanoparticles (complexes containing multiple materials, such as lipids, polymers, and peptides) for further transfection. Hybrid nanoparticles can integrate the advantages of their components, usually have a core-shell structure, and the “core” can be adjusted in response to external stimuli, which helps to promote endosome escape (108–110). At the same time, the “shell” can enhance the stability and pharmacokinetics of the nanoparticles, and endow their surface with tunable properties (111, 112). IVT-mRNA can be loaded into the “core” or absorbed into the surface of the “shell”, thus effectively transmitting, while the release spectrum may change faster in which the surface adsorbed IVT-mRNA is released.

Hoerret et al. (113) complexed IVT-mRNA which encodes galactosidase, with polycationic peptide (protamine), and then encapsulated the complex with liposomes. The resulting lipopolysaccharide protected the IVT-mRNA for longer times and demonstrated protein expression and subsequent immune response. Su et al. (108) developed biodegradable core-shell nanoparticles whose core is poly (β-amino ester) wrapped in a double phospholipid shell, which is used to deliver IVT mRNA-based vaccines *in vivo*. The pH-responsive component (poly (β-amino ester)) was selected to promote the destruction of the endosome, while the lipid surface layer (DOTAP) was selected to minimize the toxicity of the polycationic core and effectively adsorb IVT-mRNA to the surface of the nanoparticles. The resulting nanoparticles showed efficient cell uptake and cytoplasmic localization *in vitro*, low cytotoxicity, and reporter protein expression *in vivo*. Lee et al. (114) efficiently transfected IVT-mRNA into mouse cardiac fibroblasts through a heart targeting sequence fused with 9 arginine-containing peptides (CRPPR-R9) and ternary complexes containing liposomes. The mRNA transfection efficiency of CRPPR-R9/liposomes (charge ratio 20) was 44%, which was about 2 times higher than that of liposomes alone. A key challenge with direct mRNA reprogramming is the need for weeks of transfection. The repeated use of transfection agents, such as liposomes, will produce obvious toxicity. Studies have shown that one week after cardiac fibroblasts were transfected with C-Lipo containing 75% liposome (7 μ g CRPPR-R9 peptide per 1 μ g mRNA and 0.5 μ L liposome) and liposome (Lipo, 1 μ g mRNA containing 2 μ L liposome), C-Lipo caused a 25% decrease in cell viability, while Lipo caused a 90% decrease in cell viability, and the difference in cell survival rate was more significant at 2 weeks (114). Peptide-assisted mRNA transfection of cardiac fibroblasts has high efficiency and low toxicity and can be partially reprogrammed.

## Application of mRNA Vaccine Against Infectious Diseases

The development of vaccines against infectious diseases is the most effective means to control and prevent the epidemic. The development and approval of commercial vaccines are slow and unsuitable for rapidly emerging acute viral diseases, as evidenced by the outbreak of Ebola and Zika viruses in 2014-2016 and the COVID-19 in 2019-2020. mRNA vaccines show good safety in animals and have versatility for emerging infectious diseases. It can be developed rapidly and can adapt to scalable GMP production. Self-amplifying mRNA vaccines and non-replicating mRNA vaccines are the two primary mRNA vaccines against infectious pathogens. There has been a rapid increase in the number of recently published preclinical studies in these areas, as discussed below, some of these have entered human clinical trials (as shown in **Table 1**, The table summarizes the clinical trials registered at ClinicalTrials.gov).

**Table 1:** Clinical trials of mRNA vaccines against infectious diseases.

Name	Sponsoring institution	Type	Target	Phase
CV7201	CureVac AG	RNActive viral Ag mRNA	Rabies virus	Phase I (NCT02241135)
CV7202	CureVac AG	Liposome-complexed Ag mRNA	Rabies virus	Phase I (NCT03713086)
GSK3903133A	GSK	self-amplifying mRNA in cationic nanoemulsion	Rabies virus	Phase I (NCT04062669)
VAL-339851	Moderna	Nucleoside-modified viral Ag mRNA	Influenza virus(H7N9)	Phase I (NCT03345043)
VAL-506440	Moderna	Nucleoside-modified viral Ag mRNA	Influenza virus(H10N8)	Phase I (NCT03076385)
mRNA-1345	Moderna	Nucleoside-modified viral Ag mRNA	Respiratory Syncytial Virus	Phase I (NCT04528719) Phase II & III (NCT05127434)
mRNA-1647	Moderna	Nucleoside-modified viral Ag mRNA	Cytomegalovirus	Phase II (NCT04232280) Phase III (NCT05085366)
mRNA-1443	Moderna	Nucleoside-modified viral Ag mRNA	Cytomegalovirus	Phase I (NCT03382405) Phase II (NCT04917861)
mRNA-1653	Moderna	Nucleoside-modified viral Ag mRNA	Human Metapneumovirus and Human Parainfluenza Infection	Phase I (NCT04144348, NCT03392389)
mRNA-1325	Moderna	Nucleoside-modified viral Ag mRNA	Zika virus	Phase I (NCT03014089)
mRNA-1893	Moderna	Nucleoside-modified viral Ag mRNA	Zika virus	Phase I (NCT04064905) Phase II (NCT04917861)
mRNA-1944	Moderna	Nucleoside-modified viral Ag mRNA	Chikungunya	Phase I (NCT03829384)
mRNA-1388	Moderna	Nucleoside-modified viral Ag mRNA	Chikungunya	Phase I (NCT03325075)

### Self-Amplifying mRNA

Most of the self-amplifying mRNA (SAM) vaccines currently used are based on the genomes of influenza A viruses, such as Sindbis, Semliki Forest, and Venezuelan equine encephalitis viruses genomes (115–118). Genes encoding the RNA replication machinery are intact, but genes encoding structural proteins are replaced by the antigen of interest. Since antigens encoding RNAs replicate intracellularly, the SAM platform can generate large amounts of antigens from minimal doses of vaccines. Fleeton, M. N. et al. (119) proved that mice immunized with 10 μg nude SAM vaccine encoding respiratory syncytial virus fusion protein (RSVF), influenza virus hemagglutinin (HA), or amplifying virus membrane and membrane protein (PRM-E) could produce antibody response and partially protect against lethal virus attack. Annette B. Vogel. et al. (120) proved that the nude SAM vaccine encoding hemagglutinin (HA) from the H1N1 influenza virus could produce antibody response in mice by primary immunization-booster immunization and had a completed protective effect against H1N1/PR8 infection during the challenge. With the development of RNA complexing agents, the efficacy of the SAM vaccine has been significantly improved. A novel cationic nanoemulsion prepared from SAM expressing influenza virus hemagglutinin (HA) antigen [SAM (HA)] is immunogenic in ferrets. SAM (HA) induces potent functional neutralizing antibodies and cellular immune responses in mice, characterized by HA-specific CD4+cells and CD8+cells (121). Jesse H. Erasmus et al. (122). combined nanostructured lipid carriers with replicative virus RNA (RVRNA) encoding Zika virus (ZIKV) antigens, and demonstrated that a single dose (as low as 10 ng) could completely protect mice from the deadly Zika virus Sonal Saxena. et al. (123) showed that the self-amplifying rabies RNA vaccine provided adequate protection against virulent rabies at the dose of 10 μg vaccine and induced Th1 and Th2 responses. Imperial College London has launched a phase I clinical trial of novel coronavirus SARS-CoV-2. 15 volunteers will receive a single intramuscular injection of the LNP-nCoVsaRNA vaccine against 0.1/0.3μg. Starting with a low dose and then gradually increasing to a higher dose for subsequent volunteers to test the safety and derive the optimal dose. Three hundred healthy participants are expected to participate in the trial and receive two doses of the vaccine, and if the data prove, more extensive clinical trials are planned later this year.

One of the advantages of SAM vaccines is that they produce their adjuvants in the form of dsRNA structures, replication intermediates, and other motifs that may contribute to their high potency. However, the inherent nature of these PAMP may make it challenging to regulate the inflammatory characteristics or responsiveness of SAM vaccines. In addition, compared with the mRNA that does not encode the replicon gene, the SAM vaccine has a more significant restriction on the size of the insert, and the immunogenicity of the replicator protein may theoretically limit reuse.

### Non-Replicating mRNA Vaccine

The mission of the therapeutic vaccine is to retrain the specific immune response of human immunodeficiency virus (HIV)-1 (re-educate) after the interruption of antiretroviral therapy (ART) to achieve lasting control of HIV-1 replication in virus-suppressed infections. In a double-blind, placebo-controlled IIa phase multicenter study, Wesley de Jong et al. (124) studied the safety and immunogenicity of intranodal injection of HIVACAT T-cell immunogen (HTI)-TriMix vaccine. The vaccine consists of naked mRNA targeting cytotoxic T lymphocytes (CTL) of HIV-1 subdominant and conserved regions (HTI), and mRNA encoding CD40 and CD70 ligands TLR4 as adjuvants (TriMix). The vaccine is safe and well-tolerated, but it can not prove the immunogenicity of the vaccine. Jean-Pierre Routy. et al. (125) evaluated novel immunotherapy (AGS-004) based on autologous dendritic cells (AGS-004), in which all or part of the HIV-specific proliferative immune response was specific to HIV antigens presented by AGS-004 and preferential targeting of CD8+T cells.

**Figure 5:**
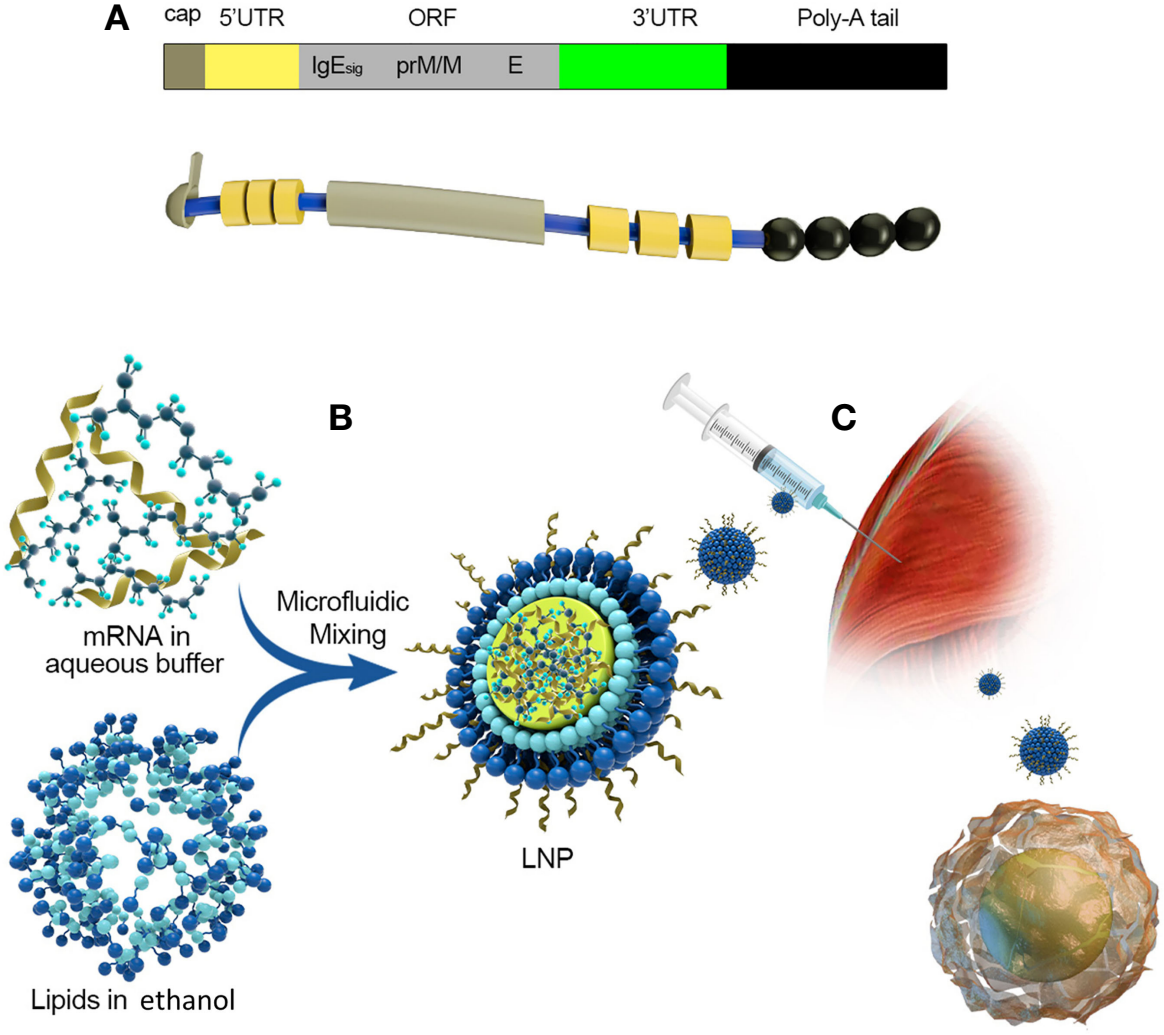
Take Moderna’s mRNA vaccine against the zika virus as an example. **(A)** The composition of mRNA:(1) cap:m7G (2) 5’UTR: Including Kozak consensus sequence (3) ORF: It consists of a signal peptide sequence and a sequence encoding PRM/M and E protein of zika virus, and 1-methyl-pseudo UTP is used instead of UTP (4) 3’UTR:3’UTR of human alpha globin (5) poly(A):30bp. **(B)** A schematic diagram of the process of encapsulating mRNA into LNPs. **(C)** Intramuscular injection and antigen presentation.

The emergence of Zika virus (ZIKV) has prompted global efforts to develop safe and effective vaccines. Justin M. Richner et al. (126) designed a modified mRNA vaccine encapsulated by LNPs that encodes wild-type or variant ZIKV structural genes and tested its immunogenicity and protection in mice. Two doses of 10μg encoding PrM-E gene containing 1-methylpseudouridine modified mRNA LNPs could produce high and low antibody titers (1100000), which had a protective effect on ZIKV infection and sterilization immunity (**Figure 5**). To eliminate the theoretical concern of ZIKV vaccine-induced cross-reaction between antibodies and related dengue virus (DENV), we designed a mutated PRM-E RNA, to destroy the conserved fusion ring epitopes in E protein. This variant protects cells or mice from ZIKV infection and reduces the production of antibodies that enhance DENV infection. Finally, the zika mRNA vaccine is also entering the clinical evaluation (NCT03014089) of phase I/II joint trial. Rabies virus glycoprotein vaccine based on mRNA has been proved to be safe and immunogenic in preclinical trials of mice and pigs (127). In phase I clinical results, 101volunteers were vaccinated with 306 doses of mRNA (80-640μg). Seven days after vaccination, subjects who received intradermal inoculation with 60/64 (94%) and intramuscular vaccination with 36/37 (97%) had injection site responses, and subjects with intradermal vaccination with 50/64 (78%) and intramuscular vaccination with 29/37 (78%) had systemic adverse events, including 10 level III events. However, the vaccine is generally safe and reasonably tolerated (128).

### mRNA Vaccines for COVID-19

The novel coronavirus (COVID-19) pandemic that emerged in December 2019, has reported more than 448 million cases worldwide and nearly 6.01 million deaths, as of March 2022 (1). In addition, COVID-19 mutates rapidly in the transmission process, and there have been reports of mutations with potential immune escape, with one or more mutations giving them worrisome epidemiological, immunological, or etiological characteristics (129). A SARS-CoV-2 vaccine is urgently needed to control the global COVID-19 public health crisis. several COVID-19 mRNA vaccines enter clinical trials (as shown in **Table 2**, The table summarizes the clinical trials registered at ClinicalTrials.gov).

**Table 2:** Clinical trials of mRNA vaccines against SARS-CoV-2.

Name	Sponsoring institution	Target	Phase	mRNA type
BNT162b2	BioNTech/Pfizer	Spike	Phase IV	Non-replicating
mRNA-1273	Moderna	Spike	Phase IV	Non-replicating
mRNA-1273.211	Moderna	Spike	Phase II/III	Non-replicating
mRNA-1273.351	Moderna	Spike	Phase I	Non-replicating
mRNA-1273.529	Moderna	Spike	Phase II	Non-replicating
CVnCoV	CureVac AG	Spike	Phase III	Non-replicating
CV2CoV	CureVac AG	Spike	Phase I	Non-replicating
MRT5500	Sanofi Pasteur and Translate Bio	Spike	Phase I/II	Non-replicating
SW0123	Stemirna Therapeutics Co., Ltd	Spike	Phase I	Non-replicating
PTX-COVID19-B	Providence Therapeutics	Spike	Phase II	Non-replicating
ChulaCov19	Chulalongkorn University	Spike	Phase I/II	Non-replicating
mRNA-1283	ModernaTX, Inc.	RBD+NTD	Phase I	Non-replicating
LVRNA009	LIVERNA THERAPEUTICS INC	RBD	Phase I	Non-replicating
DS-5670a	Daiichi Sankyo Co., Ltd.	RBD	Phase I/II	Non-replicating
ARCoV	Military Medical Research Institute/ Suzhou Aibo/Watson	RBD	Phase III	Non-replicating
ARCT-021	Arcturus/Duke-NUS	Spike	Phase II	Self-amplifying
LNP-nCOVsaRNA	ImperialCollege London/Acuitas	Spike	Phase I	Self-amplifying
HDT-301	SENAI CIMATEC	Spike	Phase I	Self-amplifying
LNP-nCOV saRNA-02 vaccine	MRC/UVRI and LSHTM Uganda Research Unit	Spike	Phase I	Self-amplifying
CoV2 SAM (LNP)	GlaxoSmithKline	Spike	Phase I	Self-amplifying
EXG-5003	Elixirgen Therapeutics, Inc	RBD	Phase I/II	Self-amplifying

Pfizer and BioNTech jointly launched an mRNA vaccine (BNT162B2), which selected the S protein of the new coronavirus as the target antigen. After several Randomized Controlled Trial (RCT) studies, it was considered to be effective for the vaccine to slow the onset of severe COVID-19, the efficiency of two doses of 30μg mRNA vaccine is more than 95% (130). In addition, the vaccine was 75% effective against the delta variant and 91% against other variants (131). Over time after full vaccination, the vaccine efficacy decreased no matter which variant strain, but there was no significant difference in the magnitude of the decrease. Pfizer has published data on serum neutralizing activity following the third dose of BNT162b2. Preliminary data show that: 6 months after the second dose, the third booster vaccine showed the same safety profile as before; for wild-type virus, the neutralizing antibody titer after the third dose was 5-8 times higher than that of the second dose; for the Beta variant (B.1.351), the neutralizing antibody titer after the third dose of vaccine was 15-21 times higher than that of the second dose. Omicron is a highly differentiated mutant of COVID-19 whose Spike contains multiple previously unseen mutation sites, and the RBD and N-terminal domains contain 15 and 8 mutation sites, respectively (132). Alexander Muik et al. (133) showed that the geometric mean neutralizing antibody titer (GMTS) of pseudovirus against omicron decreased by 22.8 times after inoculation of two doses of BNT162b2 compared with the Wuhan strain. After the third dose of the vaccine, the GMTS against omicron was 23.4 times higher than that of the second dose, similar to the GMTS of BNT162b2 inoculated twice against Wuhan pseudovirus. These data suggest that three doses of the BNT162b2 vaccine can protect against omicron-mediated COVID-19. Andrews, N., et al. (134) showed that the protective rate of two doses of mRNA vaccine BNT162b2 against omicron strain was 65.5%, significantly lower than that of 90.9% against Delta strain. Up to now, the immune protective effect after three doses of the BNT162b2 vaccine is guaranteed, even against COVID-19 mutant strains.

Kizzmekia S. Corbett. et al. (135) direct the application of atomic-level structures to pre-fusion stable mutations that improve the expression and immunogenicity of β-coronavirus spike proteins. Using this established immunogen design, the release of the SARS-CoV-2 sequence immediately triggered the rapid production of mRNA vaccine expressing pre-fusion stable the SARS-CoV-2 spike trimer (mRNA-1273). For the mRNA vaccine mRNA-1273 of Moderna, 75.1% were inoculated against omicron strain. However, when the vaccination time was more than 25 weeks, the effective rate of mRNA-1273 against omicron decreased to 14.9%, much lower than the 55.0% effective rate of the BNT162b2 vaccine (134).

Centers for disease control and prevention have assessed the adverse events of 298 million doses of COVID-19 mRNA vaccines (BNT162b2 and mRNA-1273) through VAERS and v-safe surveillance systems (from December 14, 2020, to June 14, 2021) (136). The results were shown in **Table 3**. Most of them were non-serious adverse events (92.1%), the most common of which were headache (20.4%), fatigue (16.6%), and fever (16.3%), which were common symptoms caused by the induction of an immune response in the body after vaccination (136). The research on the safety of the COVID-19 mRNA vaccine has been uninterrupted, and scientific research teams in many countries have published the results of post-marketing safety monitoring of the vaccine (137–141). So far, mRNA vaccines have an extremely high safety profile, and no serious health effects associated with mRNA vaccination have been found.

**Table 3:** The results of the mRNA vaccine safety assessment.

	Both mRNA vaccines (n=340522)	BNT162b2 vaccine (n=164669)	mRNA-1273 vaccine (n=175816)
Non-serious	313499 (92.1%)	150486 (91.4%)	162977 (92.7%)
Serious, including death	27023 (7.9%)	14183 (8.6%)	12839 (7.3%)
Serious, excluding death	22527 (6.6%)	12078 (7.3%)	10448 (5.9%)
Death	4496 (1.3%)	2105 (1.3%)	2391 (1.4%)

CureVac AG’s CVnCoV, the novel Coronavirus SARS-COV-2 lead vaccine candidate, does not use modified nucleotides in contrast to the previous two novel Coronavirus mRNA vaccines and has achieved positive preclinical results at low doses. Data showed rapid induction of a balanced immune response with a high-level viral neutralization titer (VNT) and T cell response (142). The *Phase* I dose-escalation clinical trial will include 168 healthy subjects aged 18 to 60 years with a target dose range of 2-8μg. On 16 June 2021, CureVac, a German mRNA vaccine company, announced that its phase 2B/3 HERALD trial of the mRNA vaccine developed by CureVac showed that the CVnCoV vaccine was only 47% protective against COVID-19 (143). It is also the lowest protection of any vaccine that has completed clinical trials. On 12 October 2021, CureVac, a German company, issued a notice to “stop the development of THE COVID-19 mRNA vaccine” due to the low protection rate (144).

The continuous variation of COVID-19 weakens the effectiveness of the existing vaccines on the market in preventing infection (145). Virologist Pual Bieniasz (146) suggested that it is unlikely that the vaccine will maintain its best efficacy, and it needs to be updated. The construction of mRNA vaccines against mutants by Moderna, such as mRNA-1273.211, mRNA-1273.351, and mRNA-1273.529, have entered the clinical stage. Innovative strategies for viral mutations also include the development of vaccines that target conservative sequences of the virus, or vaccines that encode broad-spectrum neutralizing antibodies, such as multivalent vaccines. Yu Liang et al. (147) innovatively designed a mutation-integrated trimerized fusion protein mutI-tri-RBD for the high variability of COVID-19 and developed a single-component broad-spectrum recombinant protein vaccine (NVSI-06-08), that is, the second-generation recombinant protein new crown vaccine. Wei et al. (148) built a circular RNA vaccine technology platform and developed circular RNA vaccines against COVID-19 and its series of variants. The circular RNA vaccine (circRNARBD-Delta) against the COVID-19 delta variant prepared in this study has broad-spectrum protection against various new coronavirus variants. The development of the COVID-19 vaccines still needs to continue.

## Conclusions

In summary, mRNA vaccines have the advantages of high safety, high efficiency, and high yield. At present, with the outbreak of covid-19, the existing BNT162b2 and mRNA-1273 have been approved for market, and 17 mRNA vaccines are under clinical research. Numerous efforts have been made to modify the structure of IVT mRNA to systematically improve its intracellular stability and translational efficiency. The research on the safety of the mRNA vaccine has been uninterrupted, and researchers in many countries have published the results that no serious health effects associated with mRNA vaccination have been found. These studies make it possible for the broader use of mRNA vaccines in the future.

## Author Contributions

LY and CL conceived and designed the framework of this article. LY and LT prepared the draft of the manuscript. MZ and CL reviewed and edited the manuscript. All the authors checked the article.

## Funding

This work was supported by the National Key Research and Development Program of China (No. 2019YFA0903801), the National Natural Science Foundation of China (No. 52073015), and Fundamental Research Funds for the Central Universities (No. ZY2006).

## Conflict of Interest

The authors declare that this review was conducted in the absence of any commercial or financial relationships that could be construed as a potential conflict of interest.

The handling editor JW declared past co-authorships with the authors CL.

## Publisher’s Note

All claims expressed in this article are solely those of the authors and do not necessarily represent those of their affiliated organizations, or those of the publisher, the editors and the reviewers. Any product that may be evaluated in this article, or claim that may be made by its manufacturer, is not guaranteed or endorsed by the publisher.

## References

[1] World Health Organization World Health Organization. Available at: http://www.who.int/.

[2] Younger DS Younger AP Guttmacher S. Childhood Vaccination: Implications for Global and Domestic Public Health. Neurol Clinics (2016) 34(4):1035–47. doi: 10.1016/j.ncl.2016.05.004 27719987

[3] Franz S. GlaxoSmithKline Malaria Vaccine Phase 3 Trial Heralded. Nat Biotechnol (2011) 29(12):1060–2. doi: 10.1038/nbt1211-1060b 22158344

[4] Calmette A. Preventive Vaccination Against Tuberculosis With BCG. Proc R Soc Med (1931) 24(11):1481–90. doi: 10.1177/003591573102401109 PMC2182232 19988326

[5] Trunz BB Fine P Dye C. Effect of BCG Vaccination on Childhood Tuberculous Meningitis and Miliary Tuberculosis Worldwide: A Meta-Analysis and Assessment of Cost-Effectiveness. Lancet (London England) (2006) 367(9517):1173–80. doi: 10.1016/S0140-6736(06)68507-3 16616560

[6] Andersen P Kaufmann SH. Novel Vaccination Strategies Against Tuberculosis. Cold Spring Harbor Perspect Med (2014) 4(6):a018523. doi: 10.1101/cshperspect.a018523 PMC4031959 24890836

[7] Andersen P Doherty TM. The Success and Failure of BCG - Implications for a Novel Tuberculosis Vaccine. Nat Rev Microbiol (2005) 3(8):656–62. doi: 10.1038/nrmicro1211 16012514

[8] Flynn NM Forthal DN Harro CD Judson FN Mayer KH Para MF. Placebo-Controlled Phase 3 Trial of a Recombinant Glycoprotein 120 Vaccine to Prevent HIV-1 Infection. J Infect Dis (2005) 191(5):654–65. doi: 10.1086/428404 15688278

[9] Pitisuttithum P Gilbert P Gurwith M Heyward W Martin M van Griensven F. Randomized, Double-Blind, Placebo-Controlled Efficacy Trial of a Bivalent Recombinant Glycoprotein 120 HIV-1 Vaccine Among Injection Drug Users in Bangkok, Thailand. J Infect Dis (2006) 194(12):1661–71. doi: 10.1086/508748 17109337

[10] Wolff JA Malone RW Williams P Chong W Acsadi G Jani A. Direct Gene Transfer Into Mouse Muscle *In Vivo* . Science (1990) 247 Pt 1):1465–8. doi: 10.1126/science.1690918 1690918

[11] Pardi N Hogan MJ Porter FW Weissman D. mRNA Vaccines - a New Era in Vaccinology. Nat Rev Drug Discovery (2018) 17(4):261–79. doi: 10.1038/nrd.2017.243 PMC5906799 29326426

[12] Sahin U Karikó K Türeci Ö. mRNA-Based Therapeutics–Developing a New Class of Drugs. Nat Rev Drug Discovery (2014) 13(10):759–80. doi: 10.1038/nrd4278 25233993

[13] Brito LA Kommareddy S Maione D Uematsu Y Giovani C Berlanda Scorza F. Self-Amplifying mRNA Vaccines. Adv Genet (2015) 89:179–233. doi: 10.1016/bs.adgen.2014.10.005 25620012

[14] Weissman D. mRNA Transcript Therapy. Expert Rev Vaccines (2015) 14(2):265–81. doi: 10.1586/14760584.2015.973859 25359562

[15] Karikó K Kuo A Barnathan E. Overexpression of Urokinase Receptor in Mammalian Cells Following Administration of the *In Vitro* Transcribed Encoding mRNA. Gene Ther (1999) 6(6):1092–100. doi: 10.1038/sj.gt.3300930 10455412

[16] Kallen KJ Theß A. A Development That may Evolve Into a Revolution in Medicine: mRNA as the Basis for Novel, Nucleotide-Based Vaccines and Drugs. Ther Adv Vaccines (2014) 2(1):10–31. doi: 10.1177/2051013613508729 24757523 PMC3991152

[17] Li Y Kiledjian M. Regulation of mRNA Decapping. Wiley Interdiscip Rev RNA (2010) 1(2):253–65. doi: 10.1002/wrna.15 PMC13110874 21935889

[18] Gallie DR. The Cap and Poly(A) Tail Function Synergistically to Regulate mRNA Translational Efficiency. Genes Dev (1991) 5(11):2108–16. doi: 10.1101/gad.5.11.2108 1682219

[19] Stepinski J Waddell C Stolarski R Darzynkiewicz E Rhoads RE. Synthesis and Properties of mRNAs Containing the Novel "Anti-Reverse" Cap Analogs 7-Methyl(3'-O-Methyl)GpppG and 7-Methyl (3'-Deoxy)GpppG. RNA (2001) 7(10):1486–95. doi: 10.1017.S1355838201014078 PMC1370192 11680853

[20] Pasquinelli AE Dahlberg JE Lund E. Reverse 5' Caps in RNAs Made *In Vitro* by Phage RNA Polymerases. RNA (1995) 1(9):957–67. PMC1369344 8548660

[21] Grudzien-Nogalska E Jemielity J Kowalska J Darzynkiewicz E Rhoads RE. Phosphorothioate Cap Analogs Stabilize mRNA and Increase Translational Efficiency in Mammalian Cells. RNA (2007) 13(10):1745–55. doi: 10.1261/rna.701307 PMC1986804 17720878

[22] Liu H Rodgers ND Jiao X Kiledjian M. The Scavenger mRNA Decapping Enzyme DcpS is a Member of the HIT Family of Pyrophosphatases. EMBO J (2002) 21(17):4699–708. doi: 10.1093/emboj/cdf448 PMC126188 12198172

[23] van Dijk E Cougot N Meyer S Babajko S Wahle E Séraphin B. Human Dcp2: A Catalytically Active mRNA Decapping Enzyme Located in Specific Cytoplasmic Structures. EMBO J (2002) 21(24):6915–24. doi: 10.1093/emboj/cdf678 PMC139098 12486012

[24] Wang Z Jiao X Carr-Schmid A Kiledjian M. The Hdcp2 Protein is a Mammalian mRNA Decapping Enzyme. Proc Natl Acad Sci United States America (2002) 99(20):12663–8. doi: 10.1073/pnas.192445599 PMC130517 12218187

[25] Kowalska J Lewdorowicz M Zuberek J Grudzien-Nogalska E Bojarska E Stepinski J. Synthesis and Characterization of mRNA Cap Analogs Containing Phosphorothioate Substitutions That Bind Tightly to Eif4e and are Resistant to the Decapping Pyrophosphatase DcpS. RNA (2008) 14(6):1119–31. doi: 10.1261/rna.990208 PMC2390807 18430890

[26] Sachs AB Davis RW. The Poly(A) Binding Protein is Required for Poly(A) Shortening and 60S Ribosomal Subunit-Dependent Translation Initiation. Cell (1989) 58(5):857–67. doi: 10.1016/0092-8674(89)90938-0 2673535

[27] Holtkamp S Kreiter S Selmi A Simon P Koslowski M Huber C. Modification of Antigen-Encoding RNA Increases Stability, Translational Efficacy, and T-Cell Stimulatory Capacity of Dendritic Cells. Blood (2006) 108(13):4009–17. doi: 10.1182/blood-2006-04-015024 16940422

[28] Mockey M Gonçalves C Dupuy FP Lemoine FM Pichon C Midoux P. mRNA Transfection of Dendritic Cells: Synergistic Effect of ARCA mRNA Capping With Poly(A) Chains in Cis and in Trans for a High Protein Expression Level. Biochem Biophys Res Commun (2006) 340(4):1062–8. doi: 10.1016/j.bbrc.2005.12.105 16403444

[29] Granados-Riveron JT Aquino-Jarquin G. Engineering of the Current Nucleoside-Modified mRNA-LNP Vaccines Against SARS-CoV-2. Biomed pharmacother = Biomed pharmacother (2021) 142:111953. doi: 10.1016/j.biopha.2021.111953 34343897 PMC8299225

[30] Gray NK Wickens M. Control of Translation Initiation in Animals. Annu Rev Cell Dev Biol (1998) 14:399–458. doi: 10.1146/annurev.cellbio.14.1.399 9891789

[31] Kozak M. At Least Six Nucleotides Preceding the AUG Initiator Codon Enhance Translation in Mammalian Cells. J Mol Biol (1987) 196(4):947–50. doi: 10.1016/0022-2836(87)90418-9 3681984

[32] Pelletier J Sonenberg N. Insertion Mutagenesis to Increase Secondary Structure Within the 5' Noncoding Region of a Eukaryotic mRNA Reduces Translational Efficiency. Cell (1985) 40(3):515–26. doi: 10.1016/0092-8674(85)90200-4 2982496

[33] Wang Z Day N Trifillis P Kiledjian M. An mRNA Stability Complex Functions With Poly(A)-Binding Protein to Stabilize mRNA In Vitro. Mol Cell Biol (1999) 19(7):4552–60. doi: 10.1128/MCB.19.7.4552 PMC84253 10373504

[34] Pascolo S. Vaccination With Messenger RNA (mRNA). Handb Exp Pharmacol (2008) 183):221–35. doi: 10.1007/978-3-540-72167-3_11 18071662

[35] Ross J Sullivan TD. Half-Lives of Beta and Gamma Globin Messenger RNAs and of Protein Synthetic Capacity in Cultured Human Reticulocytes. Blood (1985) 66(5):1149–54. doi: 10.1182/blood.V66.5.1149.1149 4052630

[36] Sahin U Holtkamp S Türeci Ö Kreiter S. Modification of RNA, producing an increased transcript stability and translation efficiency. United States patent US 9476055 (2016).

[37] Wang Z Kiledjian M. The Poly(A)-Binding Protein and an mRNA Stability Protein Jointly Regulate an Endoribonuclease Activity. Mol Cell Biol (2000) 20(17):6334–41. doi: 10.1128/MCB.20.17.6334-6341.2000 PMC86108 10938110

[38] van der Velden AW Voorma HO Thomas AA. Vector Design for Optimal Protein Expression. BioTechniques (2001) 31(3):572, 4, 6–80. doi: 10.2144/01313rr02 11570501

[39] Zinckgraf JW Silbart LK. Modulating Gene Expression Using DNA Vaccines With Different 3'-UTRs Influences Antibody Titer, Seroconversion and Cytokine Profiles. Vaccine (2003) 21(15):1640–9. doi: 10.1016/S0264-410X(02)00740-5 12639485

[40] Bergman N Moraes KC Anderson JR Zaric B Kambach C Schneider RJ. Lsm Proteins Bind and Stabilize RNAs Containing 5' Poly(A) Tracts. Nat Struct Mol Biol (2007) 14(9):824–31. doi: 10.1038/nsmb1287 17694069

[41] Chen CY Shyu AB. AU-Rich Elements: Characterization and Importance in mRNA Degradation. Trends Biochem Sci (1995) 20(11):465–70. doi: 10.1016/S0968-0004(00)89102-1 8578590

[42] Gustafsson C Govindarajan S Minshull J. Codon Bias and Heterologous Protein Expression. Trends Biotechnol (2004) 22(7):346–53. doi: 10.1016/j.tibtech.2004.04.006 15245907

[43] Zhong F Cao W Chan E Tay PN Cahya FF Zhang H. Deviation From Major Codons in the Toll-Like Receptor Genes is Associated With Low Toll-Like Receptor Expression. Immunology (2005) 114(1):83–93. doi: 10.1111/j.1365-2567.2004.02007.x 15606798 PMC1782050

[44] Ngumbela KC Ryan KP Sivamurthy R Brockman MA Gandhi RT Bhardwaj N. Quantitative Effect of Suboptimal Codon Usage on Translational Efficiency of mRNA Encoding HIV-1 Gag in Intact T Cells. PLoS One (2008) 3(6):e2356. doi: 10.1371/journal.pone.0002356 18523584 PMC2387063

[45] Van Gulck ER Ponsaerts P Heyndrickx L Vereecken K Moerman F De Roo A. Efficient Stimulation of HIV-1-Specific T Cells Using Dendritic Cells Electroporated With mRNA Encoding Autologous HIV-1 Gag and Env Proteins. Blood (2006) 107(5):1818–27. doi: 10.1182/blood-2005-01-0339 16263796

[46] Kudla G Lipinski L Caffin F Helwak A Zylicz M. High Guanine and Cytosine Content Increases mRNA Levels in Mammalian Cells. PLoS Biol (2006) 4(6):e180. doi: 10.1371/journal.pbio.0040180 16700628 PMC1463026

[47] Thess A Grund S Mui BL Hope MJ Baumhof P Fotin-Mleczek M. Sequence-Engineered mRNA Without Chemical Nucleoside Modifications Enables an Effective Protein Therapy in Large Animals. Mol Ther J Am Soc Gene Ther (2015) 23(9):1456–64. doi: 10.1038/mt.2015.103 PMC4817881 26050989

[48] Kudla G Murray AW Tollervey D Plotkin JB. Coding-Sequence Determinants of Gene Expression in Escherichia Coli (New York, Ny). Science (2009) 324(5924):255–8. doi: 10.1126/science.1170160 PMC3902468 19359587

[49] Buhr F Jha S Thommen M Mittelstaet J Kutz F Schwalbe H. Synonymous Codons Direct Cotranslational Folding Toward Different Protein Conformations. Mol Cell (2016) 61(3):341–51. doi: 10.1016/j.molcel.2016.01.008 PMC4745992 26849192

[50] Yu CH Dang Y Zhou Z Wu C Zhao F Sachs MS. Codon Usage Influences the Local Rate of Translation Elongation to Regulate Co-Translational Protein Folding. Mol Cell (2015) 59(5):744–54. doi: 10.1016/j.molcel.2015.07.018 PMC4561030 26321254

[51] Chen N Xia P Li S Zhang T Wang TT Zhu J. RNA Sensors of the Innate Immune System and Their Detection of Pathogens. IUBMB Life (2017) 69(5):297–304. doi: 10.1002/iub.1625 28374903 PMC7165898

[52] Anderson BR Muramatsu H Jha BK Silverman RH Weissman D Karikó K. Nucleoside Modifications in RNA Limit Activation of 2'-5'-Oligoadenylate Synthetase and Increase Resistance to Cleavage by RNase L. Nucleic Acids Res (2011) 39(21):9329–38. doi: 10.1093/nar/gkr586 PMC3241635 21813458

[53] Anderson BR Muramatsu H Nallagatla SR Bevilacqua PC Sansing LH Weissman D. Incorporation of Pseudouridine Into mRNA Enhances Translation by Diminishing PKR Activation. Nucleic Acids Res (2010) 38(17):5884–92. doi: 10.1093/nar/gkq347 PMC2943593 20457754

[54] Nallagatla SR Bevilacqua PC. Nucleoside Modifications Modulate Activation of the Protein Kinase PKR in an RNA Structure-Specific Manner. RNA (2008) 14(6):1201–13. doi: 10.1261/rna.1007408 PMC2390794 18426922

[55] Karikó K Muramatsu H Welsh FA Ludwig J Kato H Akira S. Incorporation of Pseudouridine Into mRNA Yields Superior Nonimmunogenic Vector With Increased Translational Capacity and Biological Stability. Mol Ther J Am Soc Gene Ther (2008) 16(11):1833–40. doi: 10.1038/mt.2008.200 PMC2775451 18797453

[56] Karikó K Muramatsu H Ludwig J Weissman D. Generating the Optimal mRNA for Therapy: HPLC Purification Eliminates Immune Activation and Improves Translation of Nucleoside-Modified, Protein-Encoding mRNA. Nucleic Acids Res (2011) 39(21):e142. doi: 10.1093/nar/gkr695 21890902 PMC3241667

[57] Fotin-Mleczek M Duchardt KM Lorenz C Pfeiffer R Ojkić-Zrna S Probst J. Messenger RNA-Based Vaccines With Dual Activity Induce Balanced TLR-7 Dependent Adaptive Immune Responses and Provide Antitumor Activity. J immunother (Hagerstown Md 1997) (2011) 34(1):1–15. doi: 10.1097/CJI.0b013e3181f7dbe8 21150709

[58] Rettig L Haen SP Bittermann AG von Boehmer L Curioni A Krämer SD. Particle Size and Activation Threshold: A New Dimension of Danger Signaling. Blood. (2010) 115(22):4533–41. doi: 10.1182/blood-2009-11-247817 20304804

[59] Pardi N Muramatsu H Weissman D Karikó K. *In Vitro* Transcription of Long RNA Containing Modified Nucleosides. Methods Mol Biol (2013) 969:29–42. doi: 10.1007/978-1-62703-260-5_2 23296925

[60] Martin SA Paoletti E Moss B. Purification of mRNA Guanylyltransferase and mRNA (Guanine-7-) Methyltransferase From Vaccinia Virions. J Biol Chem (1975) 250(24):9322–9. doi: 10.1016/S0021-9258(19)40646-7 1194286

[61] Neumann E Schaefer-Ridder M Wang Y Hofschneider PH. Gene Transfer Into Mouse Lyoma Cells by Electroporation in High Electric Fields. EMBO J (1982) 1(7):841–5. doi: 10.1002/j.1460-2075.1982.tb01257.x PMC553119 6329708

[62] Van Tendeloo VF Snoeck HW Lardon F Vanham GL Nijs G Lenjou M. Nonviral Transfection of Distinct Types of Human Dendritic Cells: High-Efficiency Gene Transfer by Electroporation Into Hematopoietic Progenitor- But Not Monocyte-Derived Dendritic Cells. Gene Ther (1998) 5(5):700–7. doi: 10.1038/sj.gt.3300626 9797876

[63] Su Z Dannull J Yang BK Dahm P Coleman D Yancey D. Telomerase mRNA-Transfected Dendritic Cells Stimulate Antigen-Specific CD8+ and CD4+ T Cell Responses in Patients With Metastatic Prostate Cancer. J Immunol (2005) 174(6):3798–807. doi: 10.4049/jimmunol.174.6.3798 15749921

[64] Wilgenhof S Van Nuffel AMT Benteyn D Corthals J Aerts C Heirman C. A Phase IB Study on Intravenous Synthetic mRNA Electroporated Dendritic Cell Immunotherapy in Pretreated Advanced Melanoma Patients. Ann Oncol Off J Eur Soc Med Oncol (2013) 24(10):2686–93. doi: 10.1093/annonc/mdt245 23904461

[65] Kyte JA Mu L Aamdal S Kvalheim G Dueland S Hauser M. Phase I/II Trial of Melanoma Therapy With Dendritic Cells Transfected With Autologous tumor-mRNA. Cancer Gene Ther (2006) 13(10):905–18. doi: 10.1038/sj.cgt.7700961 16710345

[66] Van Driessche A Van de Velde AL Nijs G Braeckman T Stein B De Vries JM. Clinical-Grade Manufacturing of Autologous Mature mRNA-Electroporated Dendritic Cells and Safety Testing in Acute Myeloid Leukemia Patients in a Phase I Dose-Escalation Clinical Trial. Cytotherapy (2009) 11(5):653–68. doi: 10.1080/14653240902960411 19530029

[67] Van Tendeloo VF Van de Velde A Van Driessche A Cools N Anguille S Ladell K. Induction of Complete and Molecular Remissions in Acute Myeloid Leukemia by Wilms' Tumor 1 Antigen-Targeted Dendritic Cell Vaccination. Proc Natl Acad Sci USA (2010) 107(31):13824–9. doi: 10.1073/pnas.1008051107 PMC2922237 20631300

[68] Wilgenhof S Van Nuffel AM Corthals J Heirman C Tuyaerts S Benteyn D. Therapeutic Vaccination With an Autologous mRNA Electroporated Dendritic Cell Vaccine in Patients With Advanced Melanoma. J immunother (Hagerstown Md 1997) (2011) 34(5):448–56. doi: 10.1097/CJI.0b013e31821dcb31 21577140

[69] Van Nuffel AM Benteyn D Wilgenhof S Corthals J Heirman C Neyns B. Intravenous and Intradermal TriMix-Dendritic Cell Therapy Results in a Broad T-Cell Response and Durable Tumor Response in a Chemorefractory Stage IV-M1c Melanoma Patient. Cancer immunol immunother CII (2012) 61(7):1033–43. doi: 10.1007/s00262-011-1176-2 PMC11028719 22159452

[70] Van Nuffel AM Benteyn D Wilgenhof S Pierret L Corthals J Heirman C. Dendritic Cells Loaded With mRNA Encoding Full-Length Tumor Antigens Prime CD4+ and CD8+ T Cells in Melanoma Patients. Mol Ther J Am Soc Gene Ther (2012) 20(5):1063–74. doi: 10.1038/mt.2012.11 PMC3345975 22371843

[71] Aarntzen EH Schreibelt G Bol K Lesterhuis WJ Croockewit AJ de Wilt JH. Vaccination With mRNA-Electroporated Dendritic Cells Induces Robust Tumor Antigen-Specific CD4+ and CD8+ T Cells Responses in Stage III and IV Melanoma Patients. Clin Cancer Res an Off J Am Assoc Cancer Res (2012) 18(19):5460–70. doi: 10.1158/1078-0432.CCR-11-3368 22896657

[72] Johansson DX Ljungberg K Kakoulidou M Liljeström P. Intradermal Electroporation of Naked Replicon RNA Elicits Strong Immune Responses. PLoS One (2012) 7(1):e29732. doi: 10.1371/journal.pone.0029732 22238645 PMC3251598

[73] Qiu P Ziegelhoffer P Sun J Yang NS. Gene Gun Delivery of mRNA *In Situ* Results in Efficient Transgene Expression and Genetic Immunization. Gene Ther (1996) 3(3):262–8. 8646558

[74] Steitz J Britten CM Wölfel T Tüting T. Effective Induction of Anti-Melanoma Immunity Following Genetic Vaccination With Synthetic mRNA Coding for the Fusion Protein EGFP.Trp2. Cancer Immunol Immunother CII (2006) 55(3):246–53. doi: 10.1007/s00262-005-0042-5 PMC11030217 16133114

[75] Aberle JH Aberle SW Kofler RM Mandl CW. Humoral and Cellular Immune Response to RNA Immunization With Flavivirus Replicons Derived From Tick-Borne Encephalitis Virus. J Virol (2005) 79(24):15107–13. doi: 10.1128/JVI.79.24.15107-15113.2005 PMC1316042 16306582

[76] Mandl CW Aberle JH Aberle SW Holzmann H Allison SL Heinz FX. *In Vitro*-Synthesized Infectious RNA as an Attenuated Live Vaccine in a Flavivirus Model. Nat Med (1998) 4(12):1438–40. doi: 10.1038/4031 9846585

[77] Thomas CE Ehrhardt A Kay MA. Progress and Problems With the Use of Viral Vectors for Gene Therapy. Nat Rev Genet (2003) 4(5):346–58. doi: 10.1038/nrg1066 12728277

[78] Leurs C Jansen M Pollok KE Heinkelein M Schmidt M Wissler M. Comparison of Three Retroviral Vector Systems for Transduction of Nonobese Diabetic/Severe Combined Immunodeficiency Mice Repopulating Human CD34+ Cord Blood Cells. Hum Gene Ther (2003) 14(6):509–19. doi: 10.1089/104303403764539305 12718762

[79] Kiem HP Allen J Trobridge G Olson E Keyser K Peterson L. Foamy-Virus-Mediated Gene Transfer to Canine Repopulating Cells. Blood (2007) 109(1):65–70. doi: 10.1182/blood-2006-04-016741 16968897 PMC1785072

[80] Si Y Pulliam AC Linka Y Ciccone S Leurs C Yuan J. Overnight Transduction With Foamyviral Vectors Restores the Long-Term Repopulating Activity of Fancc-/- Stem Cells. Blood (2008) 112(12):4458–65. doi: 10.1182/blood-2007-07-102947 PMC2597121 18684868

[81] Ferrari S Griesenbach U Shiraki-Iida T Shu T Hironaka T Hou X. A Defective Nontransmissible Recombinant Sendai Virus Mediates Efficient Gene Transfer to Airway Epithelium In Vivo. Gene Ther (2004) 11(22):1659–64. doi: 10.1038/sj.gt.3302334 15284837

[82] Malone RW Felgner PL Verma IM. Cationic Liposome-Mediated RNA Transfection. Proc Natl Acad Sci USA (1989) 86(16):6077–81. doi: 10.1073/pnas.86.16.6077 PMC297778 2762315

[83] Zohra FT Chowdhury EH Nagaoka M Akaike T. Drastic Effect of Nanoapatite Particles on Liposome-Mediated mRNA Delivery to Mammalian Cells. Anal Biochem (2005) 345(1):164–6. doi: 10.1016/j.ab.2005.06.031 16125127

[84] Zohra FT Maitani Y Akaike T. mRNA Delivery Through Fibronectin Associated Liposome-Apatite Particles: A New Approach for Enhanced mRNA Transfection to Mammalian Cell. Biol Pharm Bull (2012) 35(1):111–5. doi: 10.1248/bpb.35.111 22223346

[85] Kauffman KJ Dorkin JR Yang JH Heartlein MW DeRosa F Mir FF. Optimization of Lipid Nanoparticle Formulations for mRNA Delivery in Vivo With Fractional Factorial and Definitive Screening Designs. Nano Lett (2015) 15(11):7300–6. doi: 10.1021/acs.nanolett.5b02497 26469188

[86] Koltover I Salditt T Rädler JO Safinya CR. An Inverted Hexagonal Phase of Cationic Liposome-DNA Complexes Related to DNA Release and Delivery. Sci (New York NY) (1998) 281(5373):78–81. doi: 10.1126/science.281.5373.78 9651248

[87] Wasungu L Hoekstra D. Cationic Lipids, Lipoplexes and Intracellular Delivery of Genes. J Controlled release Off J Controlled Release Soc (2006) 116(2):255–64. doi: 10.1016/j.jconrel.2006.06.024 16914222

[88] Tros de Ilarduya C Arangoa MA Düzgüneş N. Transferrin-Lipoplexes With Protamine-Condensed DNA for Serum-Resistant Gene Delivery. Methods Eenzymol (2003) 373:342–56. doi: 10.1016/S0076-6879(03)73022-5 14714414

[89] Wolff JA Rozema DB. Breaking the Bonds: non-Viral Vectors Become Chemically Dynamic. Mol Ther J Am Soc Gene Ther (2008) 16(1):8–15. doi: 10.1038/sj.mt.6300326 17955026

[90] Wang Y Su HH Yang Y Hu Y Zhang L Blancafort P. Systemic Delivery of Modified mRNA Encoding Herpes Simplex Virus 1 Thymidine Kinase for Targeted Cancer Gene Therapy. Mol Ther J Am Soc Gene Ther (2013) 21(2):358–67. doi: 10.1038/mt.2012.250 PMC3594035 23229091

[91] Balmayor ER Geiger JP Aneja MK Berezhanskyy T Utzinger M Mykhaylyk O. Chemically Modified RNA Induces Osteogenesis of Stem Cells and Human Tissue Explants as Well as Accelerates Bone Healing in Rats. Biomaterials (2016) 87:131–46. doi: 10.1016/j.biomaterials.2016.02.018 26923361

[92] Johler SM Rejman J Guan S Rosenecker J. Nebulisation of IVT mRNA Complexes for Intrapulmonary Administration. PLoS One (2015) 10(9):e0137504. doi: 10.1371/journal.pone.0137504 26352268 PMC4564175

[93] Guan S Rosenecker J. Nanotechnologies in Delivery of mRNA Therapeutics Using Nonviral Vector-Based Delivery Systems. Gene Ther (2017) 24(3):133–43. doi: 10.1038/gt.2017.5 28094775

[94] Geall AJ Verma A Otten GR Shaw CA Hekele A Banerjee K. Nonviral Delivery of Self-Amplifying RNA Vaccines. Proc Natl Acad Sci USA (2012) 109(36):14604–9. doi: 10.1073/pnas.1209367109 PMC3437863 22908294

[95] Kauffman KJ Webber MJ Anderson DG. Materials for non-Viral Intracellular Delivery of Messenger RNA Therapeutics. J Controlled release Off J Controlled Release Soc (2016) 240:227–34. doi: 10.1016/j.jconrel.2015.12.032 26718856

[96] Zangi L Lui KO von Gise A Ma Q Ebina W Ptaszek LM. Modified mRNA Directs the Fate of Heart Progenitor Cells and Induces Vascular Regeneration After Myocardial Infarction. Nat Biotechnol (2013) 31(10):898–907. doi: 10.1038/nbt.2682 24013197 PMC4058317

[97] Turnbull IC Eltoukhy AA Fish KM Nonnenmacher M Ishikawa K Chen J. Myocardial Delivery of Lipidoid Nanoparticle Carrying modRNA Induces Rapid and Transient Expression. Mol Ther J Am Soc Gene Ther (2016) 24(1):66–75. doi: 10.1038/mt.2015.193 PMC4754552 26471463

[98] Wittrup A Ai A Liu X Hamar P Trifonova R Charisse K. Visualizing Lipid-Formulated siRNA Release From Endosomes and Target Gene Knockdown. Nat Biotechnol (2015) 33(8):870–6. doi: 10.1038/nbt.3298 PMC4663660 26192320

[99] Koch G. Interaction of Poliovirus-Specific RNAs With HeLa Cells and *E. coli* . Curr Topics Microbiol Immunol (1973) 62:89–138. doi: 10.1007/978-3-642-65772-6_4 4364181

[100] Li M Zhao M Fu Y Li Y Gong T Zhang Z. Enhanced Intranasal Delivery of mRNA Vaccine by Overcoming the Nasal Epithelial Barrier *via* Intra- and Paracellular Pathways. J Controlled release Off J Controlled Release Soc (2016) 228:9–19. doi: 10.1016/j.jconrel.2016.02.043 26941035

[101] Qiu Y Man RCH Liao Q Kung KLK Chow MYT Lam JKW. Effective mRNA Pulmonary Delivery by Dry Powder Formulation of PEGylated Synthetic KL4 Peptide. J Controlled release Off J Controlled Release Soc (2019) 314:102–15. doi: 10.1016/j.jconrel.2019.10.026 31629037

[102] McCarthy HO McCaffrey J McCrudden CM Zholobenko A Ali AA McBride JW. Development and Characterization of Self-Assembling Nanoparticles Using a Bio-Inspired Amphipathic Peptide for Gene Delivery. J Controlled Release Off J Controlled Release Soc (2014) 189:141–9. doi: 10.1016/j.jconrel.2014.06.048 24995949

[103] van den Brand D Gorris MAJ van Asbeck AH Palmen E Ebisch I Dolstra H. Peptide-Mediated Delivery of Therapeutic mRNA in Ovarian Cancer. Eur J Pharm Biopharm Off J Arbeitsgemeinschaft fur Pharmazeutische Verfahrenstechnik Evol (2019) 141:180–90. doi: 10.1016/j.ejpb.2019.05.014 31103743

[104] Udhayakumar VK De Beuckelaer A McCaffrey J McCrudden CM Kirschman JL Vanover D. Arginine-Rich Peptide-Based mRNA Nanocomplexes Efficiently Instigate Cytotoxic T Cell Immunity Dependent on the Amphipathic Organization of the Peptide. Adv Healthcare Mater (2017) 6(13):1601412. doi: 10.1002/adhm.201601412 28436620

[105] Scheel B Aulwurm S Probst J Stitz L Hoerr I Rammensee HG. Therapeutic Anti-Tumor Immunity Triggered by Injections of Immunostimulating Single-Stranded RNA. Eur J Immunol (2006) 36(10):2807–16. doi: 10.1002/eji.200635910 17013976

[106] O'Hagan DT Ott GS De Gregorio E Seubert A. The Mechanism of Action of MF59 - an Innately Attractive Adjuvant Formulation. Vaccine (2012) 30(29):4341–8. doi: 10.1016/j.vaccine.2011.09.061 22682289

[107] Samsa MM Dupuy LC Beard CW Six CM Schmaljohn CS Mason PW. Self-Amplifying RNA Vaccines for Venezuelan Equine Encephalitis Virus Induce Robust Protective Immunogenicity in Mice. Mol Ther J Am Soc Gene Ther (2019) 27(4):850–65. doi: 10.1016/j.ymthe.2018.12.013 PMC6453513 30770173

[108] Su X Fricke J Kavanagh DG Irvine DJ. *In Vitro* and *In Vivo* mRNA Delivery Using Lipid-Enveloped pH-Responsive Polymer Nanoparticles. Mol pharm (2011) 8(3):774–87. doi: 10.1021/mp100390w PMC3354687 21417235

[109] Perche F Benvegnu T Berchel M Lebegue L Pichon C Jaffrès PA. Enhancement of Dendritic Cells Transfection *In Vivo* and of Vaccination Against B16F10 Melanoma With Mannosylated Histidylated Lipopolyplexes Loaded With Tumor Antigen Messenger RNA. Nanomed nanotechnol biol Med (2011) 7(4):445–53. doi: 10.1016/j.nano.2010.12.010 21220051

[110] Clawson C Ton L Aryal S Fu V Esener S Zhang L. Synthesis and Characterization of Lipid-Polymer Hybrid Nanoparticles With pH-Triggered Poly(Ethylene Glycol) Shedding. Langmuir ACS J Surfaces Colloids (2011) 27(17):10556–61. doi: 10.1021/la202123e PMC3166210 21806013

[111] Salvador-Morales C Zhang L Langer R Farokhzad OC. Immunocompatibility Properties of Lipid-Polymer Hybrid Nanoparticles With Heterogeneous Surface Functional Groups. Biomaterials (2009) 30(12):2231–40. doi: 10.1016/j.biomaterials.2009.01.005 PMC2699891 19167749

[112] Zhang L Chan JM Gu FX Rhee JW Wang AZ Radovic-Moreno AF. Self-Assembled Lipid–Polymer Hybrid Nanoparticles: A Robust Drug Delivery Platform. ACS Nano (2008) 2(8):1696–702. doi: 10.1021/nn800275r PMC4477795 19206374

[113] Hoerr I Obst R Rammensee HG Jung G. *In Vivo* Application of RNA Leads to Induction of Specific Cytotoxic T Lymphocytes and Antibodies. Eur J Immunol (2000) 30(1):1–7. doi: 10.1002/1521-4141(200001)30:1<1::AID-IMMU1>3.0.CO;2-# 10602021

[114] Lee K Yu P Lingampalli N Kim HJ Tang R Murthy N. Peptide-Enhanced mRNA Transfection in Cultured Mouse Cardiac Fibroblasts and Direct Reprogramming Towards Cardiomyocyte-Like Cells. Int J Nanomed (2015) 10:1841–54. doi: 10.2147/IJN.S75124 PMC4358644 25834424

[115] Atkins GJ Fleeton MN Sheahan BJ. Therapeutic and Prophylactic Applications of Alphavirus Vectors. Expert Rev Mol Med (2008) 10:e33. doi: 10.1017/S1462399408000859 19000329

[116] Lundstrom K. Alphavirus Vectors: Applications for DNA Vaccine Production and Gene Expression. Intervirology (2000) 43(4-6):247–57. doi: 10.1159/000053992 11251380

[117] Schlesinger S. Alphavirus Vectors: Development and Potential Therapeutic Applications. Expert Opin Biol Ther (2001) 1(2):177–91. doi: 10.1517/14712598.1.2.177 11727528

[118] Smerdou C Liljeström P. Non-Viral Amplification Systems for Gene Transfer: Vectors Based on Alphaviruses. Curr Opin Mol Ther (1999) 1(2):244–51. 11715947

[119] Fleeton MN Chen M Berglund P Rhodes G Parker SE Murphy M. Self-Replicative RNA Vaccines Elicit Protection Against Influenza A Virus, Respiratory Syncytial Virus, and a Tickborne Encephalitis Virus. J Infect Dis (2001) 183(9):1395–8. doi: 10.1086/319857 11294672

[120] Vogel AB Lambert L Kinnear E Busse D Erbar S Reuter KC. Self-Amplifying RNA Vaccines Give Equivalent Protection Against Influenza to mRNA Vaccines But at Much Lower Doses. Mol Ther J Am Soc Gene Ther (2018) 26(2):446–55. doi: 10.1016/j.ymthe.2017.11.017 PMC5835025 29275847

[121] Brazzoli M Magini D Bonci A Buccato S Giovani C Kratzer R. Induction of Broad-Based Immunity and Protective Efficacy by Self-Amplifying mRNA Vaccines Encoding Influenza Virus Hemagglutinin. J Virol (2016) 90(1):332–44. doi: 10.1128/JVI.01786-15 PMC4702536 26468547

[122] Erasmus JH Khandhar AP Guderian J Granger B Archer J Archer M. A Nanostructured Lipid Carrier for Delivery of a Replicating Viral RNA Provides Single, Low-Dose Protection Against Zika. Mol Ther J Am Soc Gene Ther (2018) 26(10):2507–22. doi: 10.1016/j.ymthe.2018.07.010 PMC6171036 30078765

[123] Saxena S Sonwane AA Dahiya SS Patel CL Saini M Rai A. Induction of Immune Responses and Protection in Mice Against Rabies Using a Self-Replicating RNA Vaccine Encoding Rabies Virus Glycoprotein. Vet Microbiol (2009) 136(1-2):36–44. doi: 10.1016/j.vetmic.2008.10.030 19081687

[124] Jong W Leal L Buyze J Pannus P Guardo A Salgado M. Therapeutic Vaccine in Chronically HIV-1-Infected Patients: A Randomized, Double-Blind, Placebo-Controlled Phase IIa Trial With HTI-TriMix. Vaccines (2019) 7(4):209. doi: 10.3390/vaccines7040209 PMC6963294 31817794

[125] Routy JP Boulassel MR Yassine-Diab B Nicolette C Healey D Jain R. Immunologic Activity and Safety of Autologous HIV RNA-Electroporated Dendritic Cells in HIV-1 Infected Patients Receiving Antiretroviral Therapy. Clin Immunol (Orlando Fla) (2010) 134(2):140–7. doi: 10.1016/j.clim.2009.09.009 PMC2818410 19889582

[126] Richner JM Himansu S Dowd KA Butler SL Salazar V Fox JM. Modified mRNA Vaccines Protect Against Zika Virus Infection. Cell (2017) 168(6):1114–25.e10. doi: 10.1016/j.cell.2017.02.017 28222903 PMC5388441

[127] Schnee M Vogel AB Voss D Petsch B Baumhof P Kramps T. An mRNA Vaccine Encoding Rabies Virus Glycoprotein Induces Protection Against Lethal Infection in Mice and Correlates of Protection in Adult and Newborn Pigs. PLoS Negl Trop Dis (2016) 10(6):e0004746. doi: 10.1371/journal.pntd.0004746 27336830 PMC4918980

[128] Alberer M Gnad-Vogt U Hong HS Mehr KT Backert L Finak G. Safety and Immunogenicity of a mRNA Rabies Vaccine in Healthy Adults: An Open-Label, non-Randomised, Prospective, First-in-Human Phase 1 Clinical Trial. Lancet (London England) (2017) 390(10101):1511–20. doi: 10.1016/S0140-6736(17)31665-3 28754494

[129] Lou F Li M Pang Z Jiang L Guan L Tian L. Understanding the Secret of SARS-CoV-2 Variants of Concern/Interest and Immune Escape. Front Immunol (2021) 12:744242. doi: 10.3389/fimmu.2021.744242 34804024 PMC8602852

[130] Wang X. Safety and Efficacy of the BNT162b2 mRNA Covid-19 Vaccine. New Engl J Med (2021) 384(16):1577–8. doi: 10.1056/NEJMc2036242 33596350

[131] Tartof SY Slezak JM Fischer H Hong V Ackerson BK Ranasinghe ON. Effectiveness of mRNA BNT162b2 COVID-19 Vaccine Up to 6 Months in a Large Integrated Health System in the USA: A Retrospective Cohort Study. The Lancet (2021) 398(10309):1407–16. doi: 10.1016/S0140-6736(21)02183-8 PMC8489881 34619098

[132] Chen J Wang R Gilby NB Wei GW. Omicron (B.1.1.529): Infectivity, Vaccine Breakthrough, and Antibody Resistance. Chem Inform Mode (2022) 62(2):412–22. doi: 10.1021/acs.jcim.1c01451 PMC8751645 34989238

[133] Muik A Lui BG Wallisch AK Bacher M Mühl J Reinholz J. Neutralization of SARS-CoV-2 Omicron by BNT162b2 mRNA Vaccine-Elicited Human Sera. Science (2022) 375(6581):678–80. doi: 10.1126/science.abn7591 PMC9836206 35040667

[134] Andrews N Stowe J Kirsebom F Toffa S Rickeard T Gallagher E. Covid-19 Vaccine Effectiveness Against the Omicron (B.1.1.529) Variant. New Engl J Med (2022) 386(16):1532–46. doi: 10.1056/NEJMoa2119451 PMC8908811 35249272

[135] Corbett KS Edwards DK Leist SR Abiona OM Boyoglu-Barnum S Gillespie RA. SARS-CoV-2 mRNA Vaccine Design Enabled by Prototype Pathogen Preparedness. Nature (2020) 586(7830):567–71. doi: 10.1038/s41586-020-2622-0 PMC7581537 32756549

[136] Rosenblum HG Gee J Liu R Marquez PL Zhang B Strid P. Safety of mRNA Vaccines Administered During the Initial 6 Months of the US COVID-19 Vaccination Programme: An Observational Study of Reports to the Vaccine Adverse Event Reporting System and V-Safe. Lancet Infect Dis (2022) 22(6):802–12. doi: 10.1016/S1473-3099(22)00054-8 PMC8901181 35271805

[137] Barda N Dagan N Ben-Shlomo Y Kepten E Waxman J Ohana R. Safety of the BNT162b2 mRNA Covid-19 Vaccine in a Nationwide Setting. New Engl J Med (2021) 385(12):1078–90. doi: 10.1056/NEJMoa2110475 PMC8427535 34432976

[138] Klein NP Lewis N Goddard K Fireman B Zerbo O Hanson KE. Surveillance for Adverse Events After COVID-19 mRNA Vaccination. JAMA (2021) 326(14):1390–9. doi: 10.1001/jama.2021.15072 PMC8511971 34477808

[139] Witberg G Barda N Hoss S Richter I Wiessman M Aviv Y. Myocarditis After Covid-19 Vaccination in a Large Health Care Organization. New Engl J Med (2021) 385(23):2132–9. doi: 10.1056/NEJMoa2110737 PMC8531986 34614329

[140] Patone M Mei XW Handunnetthi L Dixon S Zaccardi F Shankar-Hari M. Risks of Myocarditis, Pericarditis, and Cardiac Arrhythmias Associated With COVID-19 Vaccination or SARS-CoV-2 Infection. Nat Med (2022) 28(2):410–22. doi: 10.1038/s41591-021-01630-0 PMC8863574 34907393

[141] Kharbanda EO Haapala J DeSilva M Vazquez-Benitez G Vesco KK Naleway AL. Spontaneous Abortion Following COVID-19 Vaccination During Pregnancy. JAMA (2021) 326(16):1629–31. doi: 10.1001/jama.2021.15494 PMC8427483 34495304

[142] Rauch S Roth N Schwendt K Fotin-Mleczek M Mueller SO Petsch B. mRNA-Based SARS-CoV-2 Vaccine Candidate CVnCoV Induces High Levels of Virus-Neutralising Antibodies and Mediates Protection in Rodents. NPJ Vaccines (2021) 6(1):57. doi: 10.1038/s41541-021-00311-w 33863911 PMC8052455

[143] Kremsner PG Ahuad Guerrero RA Arana-Arri E Aroca Martinez GJ Bonten M Chandler R. Efficacy and Safety of the CVnCoV SARS-CoV-2 mRNA Vaccine Candidate in Ten Countries in Europe and Latin America (HERALD): A Randomised, Observer-Blinded, Placebo-Controlled, Phase 2b/3 Trial. Lancet Infect Dis (2022) 22(3):329–40. doi: 10.1016/S1473-3099(21)00677-0 PMC8610426 34826381

[144] Gebre MS Rauch S Roth N Yu J Chandrashekar A Mercado NB. Optimization of non-Coding Regions for a non-Modified mRNA COVID-19 Vaccine. Nature (2022) 601(7893):410–4. doi: 10.1038/s41586-021-04231-6 PMC8770133 34794169

[145] Bok K Sitar S Graham BS Mascola JR. Accelerated COVID-19 Vaccine Development: Milestones, Lessons, and Prospects. Immunity (2021) 54(8):1636–51. doi: 10.1016/j.immuni.2021.07.017 PMC8328682 34348117

[146] Callaway E Ledford H. How to Redesign COVID Vaccines So They Protect Against Variants. Nature (2021) 590(7844):15–6. doi: 10.1038/d41586-021-00241-6 33514888

[147] Liang Y Zhang J Yuan RY Wang MY He P Su JG. Design of a Mutation-Integrated Trimeric RBD With Broad Protection Against SARS-CoV-2. Cell Discovery (2022) 8(1):17. doi: 10.1038/s41421-022-00383-5 35169113 PMC8847466

[148] Qu L L, Yi Z, Shen Y Lin L Chen F Xu Y. Circular RNA vaccines against SARS-CoV-2 and emerging variants. (2021) 185(10):1728–44. doi: 10.1101/2021.03.16.435594 PMC8971115 35460644

